# Double-helix optical point spread function enables real-time mesoscopic 3D functional microangiography in the living mouse brain and skull

**DOI:** 10.1038/s41467-026-71746-9

**Published:** 2026-04-13

**Authors:** Baoyuan Zhang, Shiyao Guo, Lin Tang, Yi Chen, Lukas Glandorf, Etienne Jessen, Xuyang Chang, Tian Jin, Michael Reiss, Shuxin Lyu, Qiang Fu, Hadi Amata, Wolfgang Heidrich, Chaim Glück, Dominik Schillinger, Bruno Weber, Xosé Luís Deán-Ben, Weibo Wang, Xiong Dun, Daniel Razansky, Zhenyue Chen, Quanyu Zhou

**Affiliations:** 1 Institute of Pharmacology and Toxicology, University of Zurich, Zurich, Switzerland; 2 Institute for Biomedical Engineering, ETH Zurich and University of Zurich, Zurich, Switzerland; 3 Center of Ultra-Precision Optoelectronic Instrument Engineering, Harbin Institute of Technology, Harbin, China; 4 School of Physics Science and Engineering, Tongji University, Shanghai, China; 5 Institute for Mechanics, Computational Mechanics Group, Technical University of Darmstadt, Darmstadt, Germany; 6 King Abdullah University of Science and Technology, Thuwal, Saudi Arabia

**Keywords:** Imaging and sensing, Fluorescence imaging

## Abstract

Quantitative, volumetric imaging of cerebrovascular networks and microcirculation is essential for understanding brain function. However, rapid mesoscopic 3D imaging remains challenging because of fundamental trade-offs between spatiotemporal resolution, field of view, and sensitivity to functional parameters. Here we present a mesoscopic fluorescence imaging platform featuring a double-helix phase mask for real-time, depth-resolved measurements through the intact mouse skull. The compact phase-mask design is compatible with both laser-scanning and widefield microscopy. Using multifocal laser scanning, we demonstrate real-time volumetric in vivo imaging while discriminating calvarial from cerebral vasculature across 6.6×6.6×0.8 mm^3^ volume. Beyond high-resolution structural imaging, perfusion time-to-peak values are extracted from the laser-scanning configuration while accurate flow velocity/direction information is provided via widefield tracking of fluorescently labeled cells. We demonstrate the platform’s capabilities by analyzing brain-layer-specific perfusion dynamics and vascular topology in glioma-bearing mouse brains, offering unprecedented views for probing cerebrovascular alterations in both physiological and pathological contexts.

## Abstract (web summary)

A mesoscopic fluorescence imaging platform incorporating a double-helix phase mask enables realtime 3D mapping of cerebrovascular networks and blood flow dynamics in the living mouse brain under healthy conditions and in glioma.

## Introduction

The brain is a highly vascularized organ, with its vasculature forming a complex hierarchical network that spans from large arteries/veins to fine capillaries, delivering essential oxygen and nutrients to support neuronal activity and metabolic demands^1,2^. This intricate circulatory network plays a vital role in maintaining brain function and homeostasis^3^. Vascular alterations are hallmarks of many brain disorders, including glioma^4,5^, stroke^6^, and Alzheimer’s disease^7^. Therefore, high-resolution, large-scale volumetric imaging of the cerebral vasculature is critical for advancing our understanding of cerebral physiology^8^, neurovascular coupling^9^, and the pathophysiology of multiple neurological disorders^10^. In addition, growing evidence suggests that calvarial vessels provide critical pathways linking the skull bone marrow and the brain for molecular and cellular trafficking. However, comprehensive visualization of the entire vascular architecture, with sufficient spatial resolution and functional information, remains a persistent technical challenge. Existing imaging modalities are constrained by trade-offs among spatial resolution, field-of-view (FOV), and sensitivity to functional parameters^11^, limiting their ability to fully capture the structural complexity and flow dynamics of the cerebral vasculature.

Conventional optical imaging techniques based on point-by-point scanning and detection, such as multiphoton microscopy^12,13^, offer submicron resolution but are restricted to narrow FOVs and lack full-field access to flow velocity/direction measurements. In contrast, localization-based techniques with widefield recordings have demonstrated the capability to visualize capillary-level microcirculation and quantify flow velocity/direction by localizing and tracking intravenously injected microparticles and labeled cells^14,15^. However, these approaches fail to deliver precise depth information. In a broader view, ultrasound-based^16–19^ and optoacoustic localization methods^20^ have enabled 3D visualization of the cerebral vasculature by utilizing matrix, row-column, and spherical transducer arrays. Nevertheless, the cost and complexity of the hardware design, prolonged acquisition times, and reliance on ultrasound coupling hinder broader applicability of these approaches.

Point spread function (PSF) engineering has emerged as a promising strategy to achieve depth-resolved optical imaging in super-resolution microscopy^21–24^, where axial information is encoded into the PSF shape using, e.g., a cylindrical lens or custom phase masks placed in the detection light path. A prominent example is the double-helix PSF (DH-PSF), which encodes depth into the angular rotation of two spatially separated intensity lobes. By precisely measuring the angular orientation of these lobes, the axial position of the emitter can be determined with high precision. This mechanism enables high-precision 3D localization^21,25,26^, achieving nanometer-scale localization accuracy across a FOV spanning several tens of microns. However, translating this method to mesoscopic imaging is not straightforward due to the low transmission efficiency and prominent field aberration arising from off-axis distortions and imperfections in optical components.

In this work, we built a depth-sensing module based on a customized double-helix (DH) phase mask with an 18 mm aperture size, providing uniform, high-efficiency DH-PSF generation over 800 μm axial range. By directly imposing spatial-domain constraints within an iteration-based phase retrieval framework, the proposed design yields a 3.25-fold improvement in photon efficiency over conventional Laguerre-Gaussian (LG)-based methods^27^. Additionally, to mitigate field-dependent aberrations under mesoscopic imaging conditions, we further implemented a spatially adaptive calibration strategy, which reduces depth retrieval uncertainty by 8.2-fold. When combined with multifocal laser scanning^28,29^, our system achieves a lateral resolution of 8.61 μm and an average depth estimation precision of 3.92 μm, supporting volumetric imaging over 6.6 × 6.6 × 0.8 mm^3^, with an effective penetration depth of approximately 300 μm through the intact skull. Notably, the gain in depth information is achieved without sacrificing temporal resolution. Leveraging real-time acquisition, we extracted time-to-peak (TTP) values for individual vessels, capturing dynamic perfusion patterns. By utilizing the DH phase mask in a widefield configuration, we reconstructed flow velocity/direction in both transverse and axial planes by tracking fluorescently-labeled red blood cells (RBCs), offering a comprehensive view of structural and functional organization of cortical vasculature. We extended the application of this technique to glioma-bearing mouse models, enabling volumetric visualization and quantification of tumor-associated vascular abnormalities, including increased vessel tortuosity, disrupted vascular continuity, and altered flow directionality. By enabling depth-resolved imaging over large FOVs with functional readouts, our approach holds promise for advancing fundamental studies on cerebrovascular diseases.

## Results

### 3D fluorescence mesoscopy enabled by an optimized DH-PSF design

**Fig. 1:**
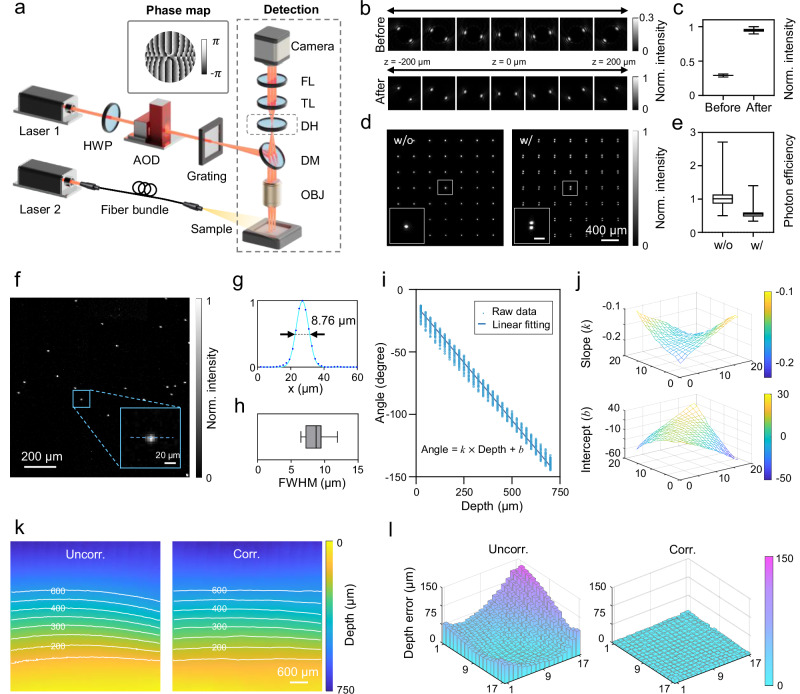
Schematic diagram and imaging performance of the DH-PSF-based fluorescence mesoscopy system. **a** Schematic of the DH-PSF-enabled 3D mesoscopy system. A customized DH phase mask is inserted at the Fourier plane of the objective to encode axial position of emitters into PSF rotation, inset shows the phase map. The detection module is compatible with both laser scanning and widefield microscopy. Abbreviations: HWP, half-wave plate; AOD, acousto-optic deflector; DM, dichroic mirror; TL, tube lens; FL, filter; OBJ, objective lens. **b** Comparison of DH-PSF intensity patterns across 39 axial planes before and after optimization. The intensity scale bar for the top row is set to 0–0.3 (before optimization), while the bottom row uses a scale of 0–1.0 (after optimization). **c** Photon efficiency for the initial LG-based design (before) and the optimized design (after), calculated across *n* = 39 axial planes. **d** Representative images of a Cy5.5 fluorescent slide under multifocal illumination at a fixed scan angle with and without the DH phase mask, together with zoom-in views (lower-left corner in both cases, scalebar: 100 μm). **e** Fluorescence intensities at each illumination spot with and without the DH phase mask (*n* = 289 illumination spots). **f** Reconstructed intensity map of DiD-labeled RBCs. **g** Line profile (interpolated) along the blue line in the inset of (**f**), with Gaussian fitting used to calculate the FWHM for lateral resolution characterization. **h** Statistical analysis of the lateral resolution from *n* = 30 randomly selected RBCs from one representative reconstructed RBC dataset. **i** Linear fit of the DH-PSF orientation as a function of depth across the entire FOV. Each spot represents data from an individual illumination spot. **j** Spatial distribution of the slope (*k*) and intercept (*b*) values extracted from the angle-depth calibration at all illumination points. **k** Reconstructed depth maps of a tilted Cy5.5 slide acquired before and after applying field-dependent calibration. White contour lines denote equal-depth planes across the FOV. **l** Depth error maps across the FOV before and after applying field-dependent calibration curve. Box plots show the median (centre line), 25-75th percentiles (box), and minimum to maximum (whiskers). Source data of (**c**, **e**, **g**, **h**, **i**, **j**, **l**) are provided as a Source Data file.

The 3D fluorescence mesoscopy platform features a depth-sensing module, which is implemented by inserting a customized DH phase mask between the objective and the tube lens (Fig. 1a). The phase mask (shown in the inset) was positioned at the back focal plane of the objective, encoding the axial position into the angular orientation of the engineered PSF lobes. The compact and modular design enables integration with different types of microscopy systems. Here we considered both laser scanning, as exemplified by multifocal illumination microscopy^30,31^, and widefield microscopy (Fig. 1a, see *Methods* for details). In the widefield mode, a collimated laser beam provides uniform epi-illumination, whereas in the laser-scanning mode, the beam is diffracted by a beam-splitting grating to generate an array of foci that are rapidly raster scanned across the sample using an acousto-optic deflector (AOD) synchronized with camera acquisition.

To adapt the DH-PSF strategy for mesoscopic imaging, we redesigned DH phase masks following an iterative phase retrieval framework with spatial-domain constraints. Specifically, the target amplitude was reshaped into a bimodal Gaussian profile. This optimization resulted in a 3.25-fold increase in photon efficiency (median 0.918 vs. 0.282) compared to the initial design based on LG modes (Fig. 1b, c). To evaluate the depth-sensing performance across the entire FOV, we employed laser scanning at a fixed scan angle and compared representative images with and without the DH phase mask (Fig. 1d). The intensity was quantified at the focal plane by integrating the signal from both lobes of the DH-PSF. Inserting the DH phase mask resulted in a photon efficiency of 54.38%, which remained highly consistent across the FOV (Fig. 1e). The DH-PSF deliberately splits the photon flux into two angularly modulated lobes, reallocating part of the conventional 2D intensity to encode axial information, a trade-off that enables robust depth localization. We next characterized the lateral resolution of the mesoscopic system by imaging DiD-labeled RBCs sparsely distributed on a microscope slide (Fig. 1f), using a 120 × 120 raster scan with a 3.3 μm step size. The lateral resolution, quantified by the FWHM of Gaussian fits to individual RBC profiles (Fig. 1g), had a median value of 8.61 μm (Fig. 1h).

To assess depth sensitivity, we imaged a Cy5.5 fluorescent slide while axially scanning across an 800 μm depth range in 25 μm steps using a motorized stage. DH-PSFs from 289 illumination spots were used to fit a linear relationship between the PSF orientation and depth (Fig. 1i). However, due to field-dependent aberrations, noticeable deviations from the fitted linear function were observed especially at the peripheral FOV. These spatial distortions compromise depth retrieval accuracy if uncorrected. To address this, we implemented a field-dependent calibration strategy. The imaging field was partitioned into a 17 × 17 grid, and for each region, local linear calibration parameters, slope (*k*) and intercept (*b*), were independently determined, followed by linear interpolation to form continuous spatial maps (Fig. 1j). Each emitter’s depth was inferred based on its PSF and the local calibration curve corresponding to its lateral position. For validation, we imaged a tilted Cy5.5 fluorescent slide, which presents a constant axial gradient along the vertical axis. The resulting isodepth contours (white lines) in the reconstructed depth map exhibit improved uniformity when using field-dependent calibration curves compared to uncorrected case (Fig. 1k). Quantitatively, the global depth error decreased from 39.17 ± 31.15 μm to 4.79 ± 1.97 μm (Fig. 1l), underscoring the importance of field-dependent correction for accurate depth retrieval in mesoscopic systems. Furthermore, a systematic Monte-Carlo simulation was conducted to assess the robustness and precision of the DH-PSF localization method against noise and photon count (Supplementary Note 1 and Supplementary Fig. 1). In addition, simulations quantifying the impact of phase-mask height profile errors confirmed the high tolerance of the DH-PSF phase mask to manufacturing defects (Supplementary Fig. 2).

Furthermore, we benchmarked our optimized DH-PSF design against a previously reported PSF engineering method that employed cylindrical lens to induce astigmatism at the mesoscale^29^ (Supplementary Fig. 3). Quantitative analysis revealed that the DH-PSF achieved a 14.9% improvement in photon transmission efficiency compared to the cylindrical lens configuration (0.54 ± 0.037 vs. 0.47 ± 0.022, mean ± standard deviation, two-sided paired *t*-test, *P* = 0.0071, *n* = 3 pairs). Moreover, the DH-PSF demonstrated enhanced depth estimation precision, as evidenced by a lower standard deviation in the fitted depth values (3.85 μm vs. 5.58 μm; *P* = 0.026, two-sided paired *t*-tests). In addition, the off-axis lobe geometry of the DH-PSF inherently reduces central overlap between adjacent emitters, unlike other PSF engineering strategies^32,33^ with centrally concentrated signals. Leveraging this structural advantage, we introduced an Alternating Direction Method of Multipliers (ADMM)-based reconstruction approach to resolve spatially overlapping PSFs under high emitter densities, as validated by phantom experiments (Supplementary Note 2 and Supplementary Figs. 4–6).

### 3D transcranial mapping of fluorescent dye perfusion in the murine brain and skull

**Fig. 2:**
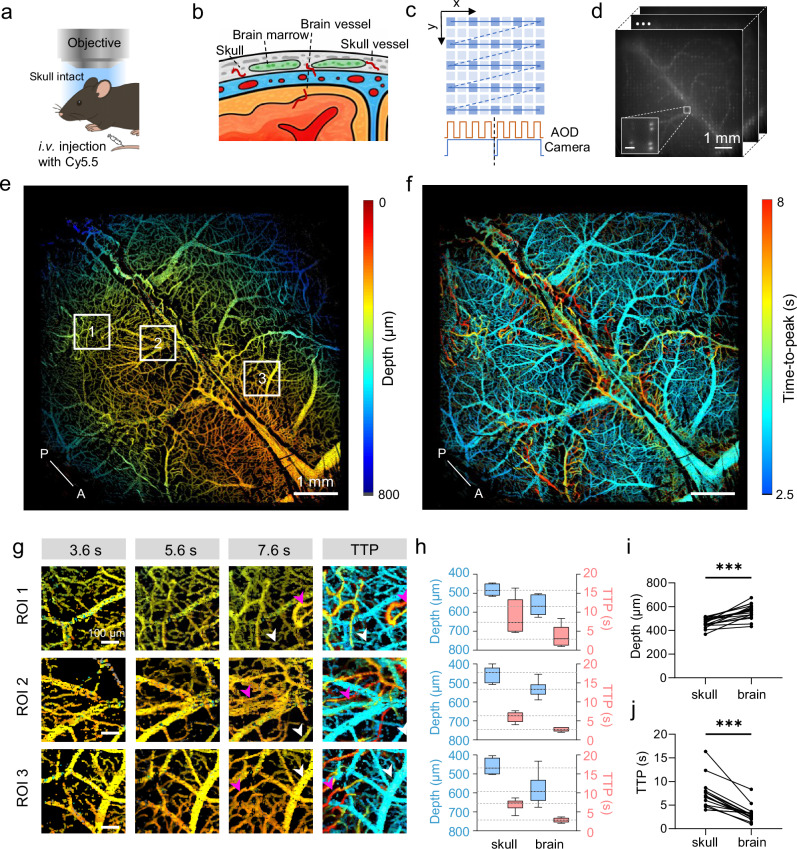
Transcranial imaging of dye perfusion patterns in various vascular compartments under laser-scanning mode. **a** Transcranial imaging of microvascular perfusion following intravenous injection of Cy5.5 dye. **b** Anatomical illustration highlighting the spatial organization between skull and brain vasculature. **c** Schematic of the multi-scan strategy and triggering scheme between the AOD and the camera. Dark squares represent the illumination lattice captured within a single camera exposure, while light squares indicate the supplemental lattice positions acquired during the multi-scan mode. **d** Raw fluorescence image captured with a zoom-in view. Representative raw frame from independent imaging experiments performed in *n* = 3 mice, with consistent results observed across animals. **e** Color-coded depth map of the microvascular network. **f** Color-coded TTP map derived from perfusion dynamics, reflecting peak arrival times of the fluorescent dye in each pixel. **g** Time-lapse images and corresponding TTP maps from three representative ROIs indicated by white boxes in **e**. Magenta arrows denote skull vessels, while white arrows indicate brain vessels. **h** Quantitative analysis of depth and TTP values for skull and brain vessels shown in (**g**). Three ROIs (labeled 1–3 in **e**) were defined in each mouse (3 mice total). Within each ROI, two paired skull-brain vessel segments were selected per mouse, resulting in *n* = 6 vessel pairs per ROI. **i**, **j** Paired measurements of the depth (**i**) and TTP (**j**) between brain and skull vessels for the same vessel pairs (*n* = 18 vessel pairs, 6 pairs per ROI, 3 ROIs per mouse, 3 mice total). Each line connects paired measurements from the same vessel pair. Statistical significance was assessed using a two-sided paired Student’s *t* test. Depth (**i**): *t*(17) = 7.243, *P* = 1.3745 × 10^−6^. TTP (**j**): *t*(17) = 7.980, *P* = 3.7764 × 10^−7^. ***P < 0.001. Box plots show the median (center line), 25–75th percentiles (box), and minimum to maximum (whiskers). Source data of (**h**–**j**) are provided as a Source Data file.

The skull bone marrow has been increasingly recognized as an active immunological niche, with calvarial vessels functioning as conduits for molecular and cellular exchange between the skull and brain^34,35^ Discriminating between skull and cortical vasculature is essential for elucidating the spatial organization and connectivity of these vascular networks. Thus, we applied the proposed DH-PSF-based methodology, combined with multifocal illumination, to capture dye-perfusion dynamics in the murine brain transcranially (Fig. 2a, b). Accurate monitoring of perfusion events across depth requires both high axial and temporal resolution. To boost the volumetric imaging speed, we implemented a multi-scan acquisition strategy, which enables scanning of 2 × 2 steps within a single camera exposure by leveraging the significantly higher scanning frequency of the AOD relative to the camera frame rate (Fig. 2c). In this configuration, the AOD operated at 4.8 kHz with a base scan grid of 20 × 20 steps. Under multi-scan mode, this was effectively expanded to a 40×40 sampling grid per volume, enabling an effective volume rate of 3 Hz (Fig. 2d).

A depth-resolved map of cerebral microcirculation in the murine brain acquired transcranially (Fig. 2e) reveals a natural axial gradient across the cortex, caused by the curvature of the skull and brain. Benefiting from the high-speed acquisition, we generated a TTP map, which highlights spatial heterogeneity in vascular perfusion timing (Fig. 2f). By jointly examining the color-coded depth and TTP maps, brain and skull vessels can be readily distinguished based on their relative depths and perfusion delays. Figure 2g shows a time-lapse image sequence (3.6 s to 7.6 s post-injection) from three representative regions of interest (ROIs) indicated in Fig. 2e, revealing distinct differences in dye arrival times among vascular compartments. Specifically, the fluorescent dye first enters the cerebral microcirculation via intracranial arteries before propagating toward the extracranial calvarial vasculature (Supplementary Movie 1). Within each ROI, a representative vessel pair was selected, one located at a deeper cortical depth (white arrow) and the other at a more superficial position, corresponding to skull vasculature (magenta arrow). Each vessel pair was quantitatively analysed for axial position and perfusion timing (Fig. 2h). Quantitative analysis across *n* = 18 paired brain-skull vessel segments from 3 mice confirmed consistent differences: brain vessels were 94.40 ± 13.09 μm (mean ± standard error of the mean) deeper than their skull counterparts (two-sided paired *t*-test, *P* = 1.3745 × 10^−6^), and exhibited 4.22 ± 0.53 s smaller TTP values (two-sided paired *t*-test, *P* = 3.7764 × 10^−7^) (Fig. 2i, j). These results demonstrate the system’s capability for unambiguous differentiation of vascular structures located at different depths, facilitating clear separation of the superficial skull vasculature from deeper brain vessels across the entire cortical surface.

### 3D multiparametric mapping of cerebrovascular dynamics in the murine brain

**Fig. 3:**
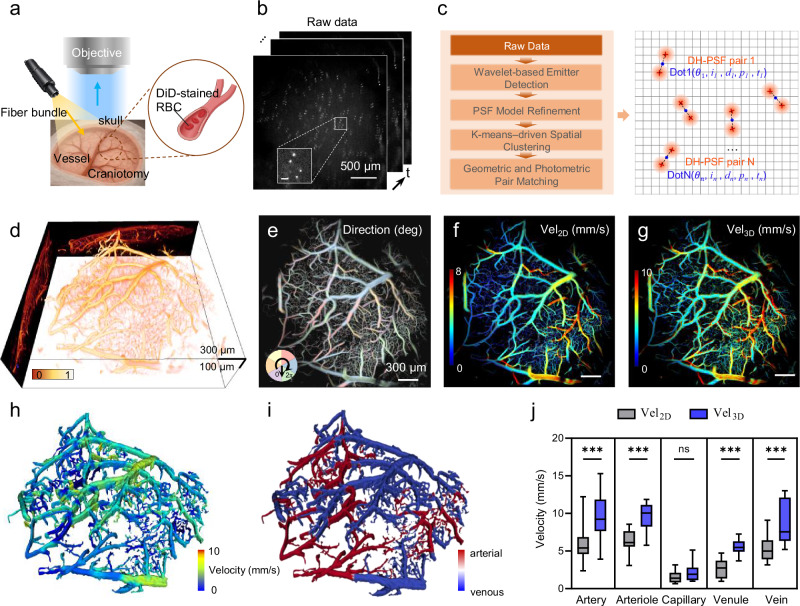
Multiparametric vascular readouts via stained RBC tracking under widefield mode. **a** Schematic of the widefield illumination configuration for in vivo brain imaging. A fiber-coupled laser was employed to uniformly illuminate the cortical surface, and DiD-labeled RBCs were intravenously injected to provide fluorescence contrast. **b** Raw fluorescence images acquired under widefield illumination. Inset: magnified view showing characteristic double-lobed DH-PSFs. Representative image from independent experiments performed in *n* = 3 mice, yielding similar results across animals. **c** Lobe pairing using an adaptive k-means clustering-based algorithm. Reconstructed RBC density map (**d**) together with color-coded direction (**e**), transverse velocity (**f**), total velocity (**g**) maps. The transverse velocity map was generated by considering only planar (xy) displacements of tracked emitters. The total velocity map incorporated both transverse and axial displacement components. **h** Vessel-averaged flow velocity overlaid on the vessel segmentation graph. **i** Artery/vein labeling. **j** Comparison of absolute Vel_2D_ (gray) and Vel_3D_ (blue) across vessel types (*n* = 10 paired vessels per vessel type from 3 mice). Vel_2D_ and Vel_3D_ were measured from the same vessels (paired measurements). Box plots show the median (centre line), 25–75th percentiles (box), and minimum to maximum (whiskers). Statistical significance was assessed using two-sided paired Student’s *t*-tests. No adjustment was made for multiple comparisons. Artery, *t*(9) = 7.304, *P* = 4.55 × 10^−5^; arteriole, *t*(9) = 9.675, *P* = 4.71 × 10^−6^; capillary, *t*(9) = 2.230, *P* = 0.0527; venule, *t*(9) = 8.423, *P* = 1.46 × 10^−5^; vein, *t*(9) = 7.099, *P* = 5.67 × 10^−5^. ns, *P* ≥ 0.05; *P < 0.05; **P < 0.01; ***P < 0.001. Source data of (**j**) are provided as a Source Data file.

The DH-PSF sensing module could also be integrated with widefield microscopy to track intravenously injected DiD-labeled RBCs in mice through a cranial window (Fig. 3a). These sparsely labeled RBCs within the vasculature act as point emitters for depth estimation, appearing as characteristic double-lobed DH-PSFs in raw images (Fig. 3b). Each recording lasted 2 min. The labeling density was empirically chosen to balance the temporal resolution required for vascular map reconstruction with the need to minimize PSF overlap. Using a lower RBC labeling density would further reduce the probability of PSF overlap but would necessitate proportionally longer recordings to capture sufficient RBC trajectories and flow information for reconstructing a vascular map of comparable quality.

In contrast to the laser-scanning mode, where the positions of scanning spots serve as prior information to facilitate the pairing of DH-PSF lobes, the widefield mode necessitated a robust lobe-clustering algorithm. Thus, we first localized the center of lobes in each frame with sub-pixel resolution, followed by the application of a k-means driven spatial clustering approach. In this step, emitters were paired based on spatial proximity, intensity similarity, and geometric constraints (Fig. 3c, see *Methods* for details). The orientation of DH-PSFs was mapped to depth for each localized RBC to obtain its 3D coordinates, which serves as the input for the 3D tracking algorithm^36^. These temporally resolved trajectories enabled the reconstruction of volumetric structural map (Fig. 3d), together with their flow directionality (Fig. 3e) and velocity (Fig. 3f, g) by calculating the emitter displacement between the consecutive frames in 3D. Conventional vascular velocity maps, typically derived from planar inter-frame displacements of tracked particles, yield 2D-projected velocity (Vel_2D_) fields. By incorporating full 3D inter-frame displacements of emitters, we generated both Vel_2D_ (Fig. 3f) and Vel_3D_ (Fig. 3j) maps, considering both transverse and axial velocities. The 3D structural and flow direction maps also facilitate vessel segmentation and vessel type classification. The intensity map of vasculature was first segmented using an Otsu thresholding and morphological closing. Arterial and venous identities were then recognized based on their distinct topological propagation along the vascular graph^37,38^. This framework yielded branch-averaged maps of flow velocity (Fig. 3h), and vessel type labeling (Fig. 3i) even when pial vessels cross in close proximity. Apparent direct contact between pial arteries and veins appears when one of them dives underneath the other. Comparative analyses across vessel types (*n* = 10 paired vessels per type) demonstrated significant discrepancies between Vel_2D_ and Vel_3D_ in arteries, arterioles, venules and veins (all *P* < 0.001), but not in capillaries (*P* = 0.0527; two-sided paired *t*-tests; Fig. 3j). The magnitude of the velocity underestimation was pronounced in vessel types such as arterioles and venules, aligning with their greater tendency for steep axial inclinations^39,40^. The accuracy of axial velocity estimation is tightly linked to axial resolution. At a frame rate of 200 Hz, the axial localization uncertainty of ~4 μm corresponds to an axial velocity uncertainty of ~0.8 mm/s. Reported axial velocities in the murine brain range from 0 to 12 mm/s^41,42^. For velocities on the higher end of this range, such uncertainty corresponds to ~6.7% relative deviation, and its impact becomes more pronounced for vessels exhibiting lower axial velocities. Consequently, improving axial velocity estimation will fundamentally require enhancing axial localization precision.

**Fig. 4:**
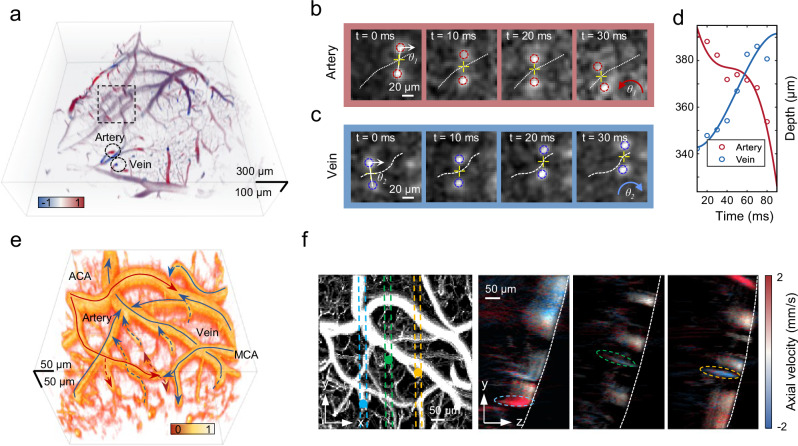
Differentiation of penetrating arterioles and venules and their axial flow dynamics. **a** Axial velocity map computed from tracked RBC trajectories, overlaid on the 3D vascular structure. Red and blue indicate upward and downward flow directions, respectively. Two representative penetrating vessels (black circles) were selected for further analysis. **b**, **c** Time-lapse DH-PSF images in the selected penetrating artery and vein. Opposite angular rotations reflect inversed flow directions. **d** Time-depth traces derived from selected penetrating artery and vein. **e** Magnified view of the ROI indicated with black box in **a**, showing color-coded flow directions (red: arterioles; blue: venules). Dashed outlines indicate penetrating vessel segments. ACA, anterior cerebral artery; MCA, middle cerebral artery. **f** Orthogonal yz projections extracted from selected slices, highlighting localized axial flow components. Source data of (**d**) are provided as a Source Data file.

Leveraging functional readouts, such as flow velocity and direction, we could further distinguish vertically oriented arterioles and venules spanning across cortical depths (Supplementary Movie 2). This was accomplished by depicting their axial velocity components with a diverging colormap (Fig. 4a), where red and blue denote upward and downward flow, respectively. Two representative penetrating vessels, one arteriole and one venule, marked with black circles in Fig. 4a, were selected for comparative analysis of axial flow characteristics. Time-lapse DH-PSF images of RBCs flowing inside vessels (Fig. 4b, c and Supplementary Fig. 7) exhibited opposite lobe rotations, reflecting their opposing flow directions. These observations were further validated by time-depth plots (Fig. 4d and Supplementary Fig. 7). A magnified ROI is shown in Fig. 4e, emphasizing the anatomical and functional context of the local vasculature. Based on the flow direction map and automated A/V labeling, vascular compartments were labeled and annotated using distinct colors and line styles (red solid line: pial artery; blue solid line: pial vein; red dashed line: penetrating artery; blue dashed line: penetrating vein), aiding in the identification of vessel type and orientation. The corresponding spatiotemporal dynamics of RBC flow within this ROI are visualized in Supplementary Movie 3, which presents the trajectories of individual particles tracked across consecutive frames, highlighting directional flow and vessel-specific transport. Orthogonal y-z slices (Fig. 4f) depict localized axial velocities, demonstrating the system’s ability to resolve both vascular geometry and depth-resolved hemodynamics of cortical microvasculature.

### Morphological and functional alterations in tumor-associated vascular remodeling

Access to both morphological and functional readouts make the proposed method a powerful tool for studying vascular alterations during pathological progression, such as during glioblastoma development. Glioblastoma is the most aggressive primary brain tumor^43,44^, characterized by rapid growth and extensive vascular proliferation. Understanding its vascular remodeling is critical for developing effective therapeutic strategies targeting the tumor microenvironment and mitigating disease progression.

**Fig. 5:**
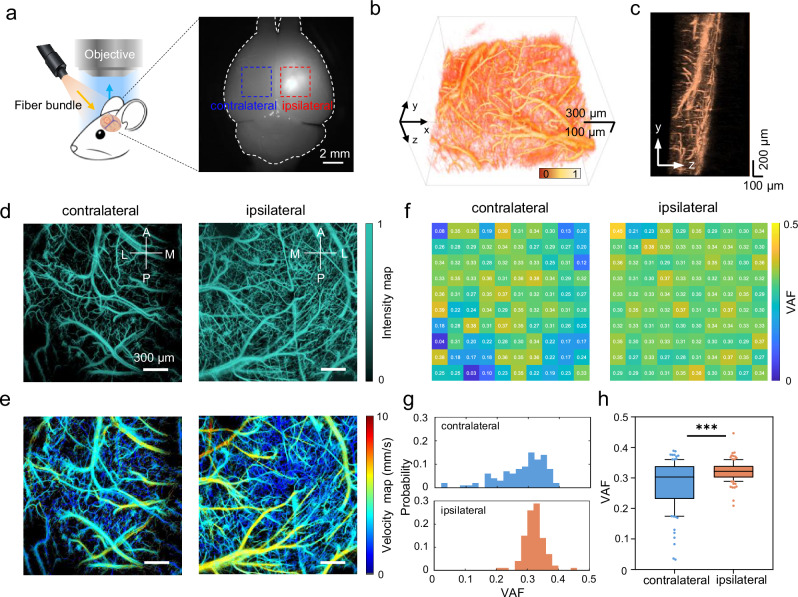
Morphological mapping of glioma-associated vascular remodeling. **a** Schematic of the experimental setup. The enlarged view illustrates the widefield fluorescence image of ex vivo mouse brain, with the red dashed box indicating the ipsilateral side and the blue box denoting the contralateral side. **b** Reconstructed 3D vasculature of the ipsilateral side. **c** yz-plane projection of (**b**). DH-PSF-derived structural (**d**) and velocity (**e**) maps of the contralateral and ipsilateral sides. **f** VAF maps over 10 × 10 subregions on both sides. **g** Probability distribution histograms of VAF values for contralateral (top) and ipsilateral (bottom) regions. **h** Statistical comparison of VAF between the ipsilateral and contralateral sides. VAF was computed over 10 × 10 subregions per side for each mouse and pooled across 3 mice (*N* = 300 subregions per group, 100 subregions per mouse). Box plots show the median (centre line), 25–75th percentiles (box), and minimum to maximum (whiskers). Statistical significance was assessed using a two-sided Mann-Whitney *U* test (no adjustment for multiple comparisons), *U* = 36225, *P* = 3.58 × 10^−5^ (approx.). Medians: ipsilateral = 0.3347, contralateral = 0.3173. ***P < 0.001. Source data of (**f**–**h**) are provided as a Source Data file.

To investigate vascular alterations associated with glioblastoma progression, we used an orthotopic brain tumor model generated via stereotactic injection of U87-MG cells in the right hemisphere of the mouse brain. Prior to functional imaging, high-resolution T1-weighted anatomical reference images were acquired using a 2D fast low angle shot (FLASH) sequence (Supplementary Fig. 8). Cortical blood flow was recorded at 100 Hz under widefield illumination 5 weeks post tumor cell injection, following skull thinning and intravenous injection of fluorescently labeled RBCs. Figure 5a illustrates the experimental setup and displays a transverse view of the cortex excised after imaging. The ipsilateral side (tumor-bearing hemisphere) exhibited localized hyperfluorescence (red box), arguably indicative of increased vascular permeability and dye extravasation^45^, whereas the contralateral (non-tumor) hemisphere, lacking such leakage, served as an internal control (blue box). The 3D vascular map of the ipsilateral side revealed a densely interconnected vascular network (Fig. 5b) while the corresponding yz-projection (Fig. 5c) highlighted the distribution of penetrating vessels within the tumor area. Structural (Fig. 5d) and velocity (Fig. 5e) maps of the contralateral (healthy) and ipsilateral (tumor) sides demonstrated a dense, disorganized vascular network with irregular branching patterns on the ipsilateral side. To quantitatively assess vascular density, both ipsilateral and contralateral ROIs were subdivided into 10×10 subregions of equal size. The vessel area fraction (VAF) was calculated for each subregion, generating spatial heatmaps (Fig. 5f). To further characterize the distribution of VAF values, probability histograms were plotted for contralateral (Fig. 5g, top) and ipsilateral (Fig. 5g, bottom) ROIs. Statistical comparison of VAF between the ipsilateral and contralateral hemispheres (Fig. 5h) showed a significant increase on the ipsilateral side (Mann-Whitney *U* test, two-sided; *n* = 3 mice; *N* = 300 ROIs per group; *U* = 36225; *P* = 3.58 × 10^−5^ (approx.)). The median VAF was higher ipsilaterally than contralaterally (0.3347 vs. 0.3173). The observed increase in median VAF is indicative of enhanced angiogenesis and vessel proliferation, likely driven by the tumor’s metabolic demands and the resulting hypoxic microenvironment^46,47^.

**Fig. 6:**
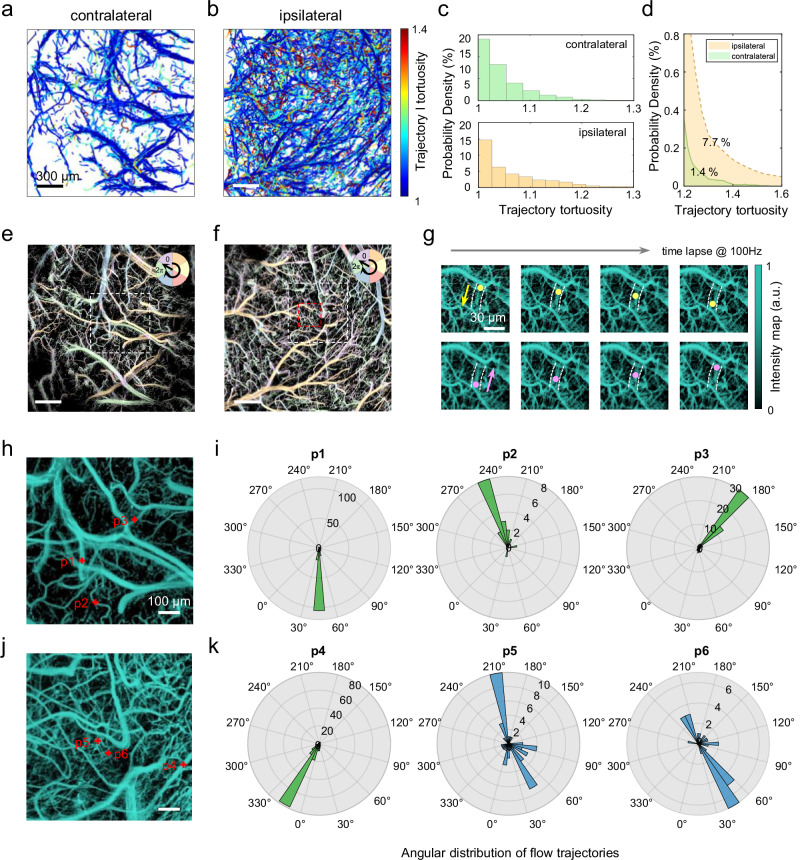
Trajectory-resolved analysis of vascular tortuosity and bidirectional flow in glioma microvasculature. **a**, **b** Color-coded RBC trajectory maps of contralateral and ipsilateral sides. **c** Tortuosity histogram of both sides, with zoom-in view in (**d**). **e**, **f** Direction maps of both sides. **g** Time-lapse example showing bidirectional flow within a vessel segment in the ipsilateral side. **h** Locations of three representative vessel segments (p1–p3) in the contralateral hemisphere (white box in **e**). **i** Polar histograms of trajectory orientations for the contralateral segments p1–p3, showing single-peaked, unidirectional flow patterns. **j** Locations of three representative vessel segments (p4–p6) in the ipsilateral side (white box in **f**) selected for angular analysis. **k** Polar histograms of trajectory orientations: unidirectional flow in p4 (left) and bidirectional peaks ~180° apart in p5 and p6 (middle and right). Source data of (**c**, **d**) are provided as a Source Data file.

One distinct advantage of the tracking-based method over traditional static imaging is its ability to capture RBC trajectories, which is utilized for subsequent analysis of vascular tortuosity and flow directionality. Vascular tortuosity was defined as the ratio of total trajectory length to the straight-line distance between its endpoints. Figure 6a, 6b show color-coded tortuosity maps in the contralateral and ipsilateral hemispheres, respectively, where ipsilateral side trajectories appeared notably more convoluted^48,49^. This observation was then confirmed quantitatively by a lower fraction of near-linear paths (tortuosity ≈ 1.0) and a higher incidence in the 1.2–1.6 range (7.7% vs. 1.4%; Fig. 6c, d) in the ipsilateral side. Individual mouse data detailing the shift in the proportion of highly tortuous trajectories is presented in Supplementary Fig. 9. We next assessed flow directionality by computing the orientation of individual trajectories. Direction maps (Fig. 6e, f) revealed highly variable flow directions in tumor vessels, in contrast to the more coherent patterns in the contralateral side. Notably, we observed counter-propagating flow, with instances where RBCs traveled in opposite directions within the same vessel segment. This phenomenon was predominantly observed in narrow, tortuous vessels^50,51^, and is exemplified in a time-lapse sequence (Fig. 6g) showing two labeled RBCs flowing in opposite directions through a shared vascular path (Supplementary Movie 4). To further investigate flow directionality, we performed angular trajectory analysis on both contralateral and ipsilateral hemispheres. On the contralateral side, three representative vessel segments (Fig. 6h) displayed unidirectional flow, as evidenced by the single-peaked angular distributions in their polar histograms (Fig. 6i). In contrast, in the ipsilateral side, three representative vessel segments (Fig. 6j) exhibited more complex flow patterns. In a middle-sized vessel (segment p4, diameter = 25.2 µm), a single dominant flow direction was observed (Fig. 6k, left), whereas segments p5 and p6 (Fig. 6k, middle and right) showed pronounced bidirectional flow with peaks spaced approximately 180° apart. Such irregular flow behaviors were absent in the contralateral control regions and may reflect impaired perfusion and loss of vascular polarity, known hallmarks of glioma-associated microcirculatory dysfunction^51,52^. These findings highlight the capability of DH-PSF enabled imaging platform to resolve alterations in vascular phenotypes associated with tumor-driven vascular remodeling.

## Discussion

In this study, we developed a depth-encoded fluorescence mesoscopy platform based on DH-PSF, capable of high-resolution, high-speed, volumetric imaging of cerebral and calvarial vasculature across large cortical fields in vivo. We designed a compact, high-throughput phase mask that converts point sources into angularly modulated DH-PSFs, enabling robust depth extraction through lobe orientation analysis. By integrating two complementary illumination modes, we achieve flexible trade-offs between spatial resolution, temporal throughput, and signal density. The phase mask is positioned in the detection path, providing a compact, lightweight, and cost-effective solution with minimal optical complexity. This contrasts sharply with more complex PSF engineering systems that rely on cascaded or multiplexed optics, often at the expense of light throughput, phase-mask size constraints, or PSF interpretability due to overlapping signals. In addition, unlike conventional PSFs that concentrate energy into a diffraction-limited core, the double-helix PSF deliberately redistributes part of the optical energy into two rotating lobes to encode axial information. While this reduces peak intensity, the broadened energy distribution provides additional intensity sampling across neighboring pixels, which improves the robustness of subpixel localization by mitigating pixel noise and interpolation errors during lobe fitting. To further address spatially and axially overlapping DH-PSFs under high-density conditions, we implemented an ADMM-based reconstruction framework that formulates emitter localization as a sparsity-constrained inverse problem (Supplementary Fig. 4–6). By leveraging the angular encoding properties of the DH-PSF, the algorithm disentangles spatially entangled emitters and enables accurate recovery of their 3D positions from overlapped observations.

Compared to other 3D imaging modalities, our DH-PSF-based strategy offers a distinct balance between large FOV, high spatiotemporal resolution, and functional imaging capability. Two-photon microscopy, while providing excellent spatial resolution and molecular specificity, suffers from a limited FOV and requires invasive craniotomy procedures, making it suboptimal for mesoscopic applications^53,54^. Ultrasound localization microscopy can access deep tissue and achieve super-resolution via sparse microbubble tracking. However, its spatial resolution is still influenced by acoustic diffraction and is particularly prone to skull-induced aberrations in transcranial imaging^55,56^. Optical coherence microscopy offers label-free flow estimation based on Doppler effect but is mainly sensitive to motion along A-scans and requires minutes to image large regions in the mouse brain^57,58^. Our mesoscopic approach fills this critical gap by combining optical-resolution, large-area coverage, and depth-resolved flow analysis through an intact skull at high frame rates, without the need for complex beamforming, acoustic coupling, or mechanical scanning. A performance comparison between the proposed method and other state-of-the-art mesoscopic imaging techniques is shown in Supplementary Table 1.

Our findings not only validate the system’s capacity to resolve depth-dependent perfusion dynamics, but also underscore its potential to address biologically significant questions that remain elusive with conventional imaging modalities. By combining volumetric access with functional sensitivity, the platform offers a powerful tool for investigating pathological conditions characterized by disrupted hemodynamic pathways. We validated the system’s utility in a glioma model, where depth-resolved flow mapping revealed heterogeneous vascular remodeling and aberrant microcirculatory patterns in tumor-involved regions. These observations suggest perfusion heterogeneity in tumor-associated vasculature, illustrating the platform’s potential to resolve tumor-related hemodynamic phenotypes in vivo. The current study focuses on vascular imaging since the DH phase mask was optimized at a central wavelength of ~700 nm, which is well suited for far-red fluorescent dyes used in vascular labeling. The DH-PSF platform can potentially be applied to image other brain structures, e.g. neurons, astrocytes, or glial cells, provided that the excitation and design wavelengths are matched and suitable labeling strategies are implemented. In widefield imaging mode, sparse labeling is required to keep individual emitters distinguishable from surrounding emitters, which can be achieved through diluted viral transfections or cell type-specific Cre or CreER driver mouse lines^59^. In laser-scanning imaging mode, sparse labeling is not strictly required, though reduced labeling density may be beneficial for enhancing image contrast. Thus, conventional genetically encoded fluorescent proteins can be used to label cell populations more densely than in widefield imaging mode.

Despite these advantages, the system has several limitations. In the laser-scanning mode, even with multi-scan acquisition, the sparse excitation pattern results in inefficient utilization of the camera’s sensor area, as many pixels remain unexposed or unused during each frame. This could potentially be addressed by incorporating complementary encoding strategies, such as wavelength multiplexing^60^ to increase effective data density per frame. For example, two different wavelengths could be combined on the illumination side, and a beam-splitting grating could be used to introduce a lateral displacement between their projected patterns on the camera, thereby doubling the utilization efficiency of the camera pixels. Furthermore, while DH-PSFs provide continuous depth encoding, their performance still depends on precise calibration and remains sensitive to field-dependent aberrations. Future work may focus on integrating spectral contrast for multi-chromophore imaging and implementing deep learning (DL)-based reconstruction frameworks to replace the current k-means clustering and ADMM pipeline, which could enable faster and more robust emitter localization under high-density or low-SNR conditions^61,62^. A major challenge in implementing DL-based methods is the lack of paired DH raw data and experimentally acquired ground truth, motivating the use of synthetic datasets with perfectly paired inputs and labels. This requires accurately modeling depth- and wavelength-dependent PSF variations, asymmetric lobe morphology, and realistic noise levels to ensure dataset representativeness and model generalizability.

In summary, we have introduced a compact, cost-efficient, and high-resolution volumetric imaging platform based on double-helix PSF engineering for minimally invasive, capillary-scale mapping of cortical microcirculation in vivo. By concurrently resolving fluorescence intensity, depth, flow velocity, and direction across a large FOV with sub-second temporal resolution, this method enables comprehensive characterization of baseline hemodynamics, vascular architecture, and dynamic perfusion patterns. The approach is well suited for chronic imaging in awake animal models to investigate cerebrovascular remodeling, tumor angiogenesis, and long-term adaptations.

## Methods

### DH-PSF based fluorescence mesoscopy

A customized 3D fluorescence mesoscope was developed (Fig. 1a) to support both laser scanning and widefield illumination modes, allowing flexible switching between laser-scanning and widefield imaging configurations. Both configurations share the same detection arm that incorporates a DH phase mask positioned at the exit pupil of the objective lens (CLS-SL, EFL = 70 mm, Thorlabs, USA). Fluorescence emission is collected by the objective, passed through a dichroic mirror (F38-663, Semrock, USA) and DH phase mask, and then focused by a tube lens onto a high-speed CMOS camera (1008 × 1008 pixels, 4.4 kHz at full resolution, pco.dimax S1, PCO AG, Germany). A long-pass emission filter (LP02-671RU-25, Semrock, USA) was placed in front of the camera to block residual excitation light. The magnification ratio of the imaging system was adjusted between 1.50× and 2.86× to accommodate different imaging targets, using two interchangeable tube lenses: TL1 (AF Micro-Nikkor 105 mm, Nikon, Japan) and TL2 (TTL200MP2, Thorlabs, USA).

Widefield illumination was provided by a 640 nm continuous-wave (CW) laser (MDL-HD-640, CNI, China). The laser beam was delivered via a multimode fiber and collimated to ensure uniform illumination on the sample. For brain imaging experiments, the measured illumination power at the sample plane was 139.2 mW. Laser-scanning system was implemented using a 660 nm CW laser (gem 660–500 mW, Laser Quantum, USA). The beam was expanded 4× using a relay lens pair (ACN254-050-A and AC254-200-A, Thorlabs, USA), and its polarization angle was adjusted with a half-wave plate (AHWP10M-600, Thorlabs, USA). Beam steering was performed using a two-dimensional acousto-optic deflector (2D AOD, DTSXY-400-532, AA Opto-Electronic, France), which dynamically scanned the beam across the sample plane. The steered beam was directed onto a 17×17 beam-splitting diffractive optical element (DE-R 243, Holoeye Photonics AG, Germany), producing multiple beamlets with an angular separation of 0.27°. This grid density reflects a practical balance between maintaining adequate SNR for each illumination spot and achieving sufficient spatial sampling to model field-dependent aberrations. Synchronization between the AOD and the camera was achieved via external triggering using a data acquisition board (NI PCIe-6536b, National Instruments, USA). These beams were reflected by the dichroic mirror and focused by the objective lens, forming a 2D lattice pattern on the sample. For brain imaging, the total illumination power across the illumination grid was measured at 24.6 mW, corresponding to an average power of 0.085 mW per individual illumination spot.

### Design of DH phase masks

To improve energy confinement in the main lobes and enhance the axial fidelity of DH-PSFs, we implemented an iterative phase retrieval framework that integrates a modified amplitude constraint, as illustrated in Supplementary Fig. 10.

We first construct an initial DH-PSF constructed from an equal-weight superposition of LG modes (*l*,*p*), with a fixed amplitude (*A* = 1) and phase determined by the LG model. While this initial estimate provides the desired dual-lobe rotation, it exhibits low transmission efficiency and rapid attenuation.

To address these limitations, we defined a double-peaked Gaussian amplitude distribution as the target intensity profile across 39 discrete axial planes. The iterative phase optimization process begins with the initial LG-based DH beam, which is numerically propagated to defocus plane ${z}_{k}$ (for $k$ = 1, 2, …, 39) using Fourier transform ($F$). At each defocus plane ${z}_{k}$, the resulting complex amplitudes $u\left({z}_{1}\right),\ldots,u\left({z}_{39}\right)$ are computed

1 $$u({z}_{k})={F}^{-1}[H({z}_{k})\cdot F({e}^{i{\phi }_{0}})],$$

where $H$ refers to the propagation transfer function,$\,{\phi }_{0}$ denotes the initial phase distribution at the plane $z$ = 0.

At each plane ${z}_{k}$, a target complex amplitude $u{\prime} ({z}_{k})$ is constructed, where the amplitude is defined as the square root of a double-peaked Gaussian function, i.e.,

2 $$|u{\hbox{'}}({z}_{k})|=\sqrt{{G}_{1}(x,y;\sigma )+{G}_{2}(x,y;\sigma )}$$

Each ${G}_{i}$ (for *i* = 1 and 2) represents a single 2D Gaussian distribution centered at the expected lobe positions as

3 $${G}_{i}\left(x,y,\sigma \right)=\exp \left(-\frac{{\left(x-{x}_{i}\right)}^{2}+{\left(y-{y}_{i}\right)}^{2}}{2{\sigma }^{2}}\right),$$

where$\left({x}_{i},{y}_{i}\right)$ denote the coordinates of the two main lobes in the original complex amplitude, and *σ* is the standard deviation, optimized to ensure convergence and efficiency.

The amplitudes of $u\left({z}_{1}\right),\ldots,u\left({z}_{39}\right)$ are replaced with $u{\prime} \left({z}_{1}\right),\ldots,u{\prime} \left({z}_{39}\right)$, while keeping the original phase distribution. An inverse Fourier transform (${F}^{-1}$) is then applied to retrieve the updated phase distribution in the pupil plane as

4 $${\phi }_{{new}}={\arg }\left({F}^{-1}\left\{{u}^{{\prime} }\left(z\right)\right\}\right).$$

In each iteration, the complex amplitudes obtained across the 39 planes are superposed with equal weights, and the resulting beam is normalized in amplitude before being fed into the next round of optimization. The amplitude correction constraint is enforced by comparing the current amplitude with the predefined double-peak Gaussian function. Through successive iterations, the beam profile progressively converges toward the desired axial and lateral intensity distribution. Upon convergence, the final phase-only mask is extracted and used for fabrication. To accommodate varying imaging depths and optical architectures, we designed three phase masks, each featuring an aperture size of 18 mm. Detailed design specifications are provided in Supplementary Table 2.

### Fabrication of DH phase masks

The DH phase masks were fabricated on a fused silica wafer using a combination of multi-level i-line photolithography and reactive-ion etching (RIE). The process utilized a sputtered Cr film (150 nm) as a hard mask. Each lithographic cycle involved hexamethyldisilazane (HMDS) promotion, AZ1505 spin-coating, alignment (1 μm accuracy), and 9 mJ/cm^2^ UV exposure using an i-line contact aligner. Pattern transfer into the fused silica was achieved through four sequential RIE cycles using CHF_3_ (15 sccm) and O_2_ (5 sccm) plasma. The cumulative etch depths of 96 nm, 192 nm, 384 nm, and 768 nm yielded the final 16-level diffractive-holographic phase structure, with a maximum height of 1.5374 μm. The step-by-step fabrication procedures are included as Supplementary Table 3.

### Calibration of multifocal laser-scanning pattern

Prior to image acquisition, the exact positions of the multifocal laser-scanning pattern, comprising 289 excitation foci at each scanning angle, were calibrated. This was achieved by temporarily removing the DH phase mask from the detection path and placing a Cy5.5-labeled fluorescent reference slide at the focal plane. For each frame, a 2D Gaussian smoothing filter was applied to the acquired image to reduce noise, and the center of the illumination spots was localized using intensity-weighted centroid localization.

### Image reconstruction

An open-source particle tracking software TrackNTrace (TNT, version 1.03)^63^, originally developed for single-molecule localization microscopy, was used to localize the main lobes of DH-PSF with sub-pixel resolution. Initial emitter detection and position refinement were performed using wavelet-based multi-scale filtering followed by Gaussian fitting. This software outputs a coordinate set of spot candidates at each subframe, which serves as input for subsequent 3D reconstruction.

For datasets acquired under laser-scanning mode, the positions of the 289 excitation foci (obtained during the calibration process) were used as prior information. At each illumination spot, a square ROI with a window size of 45 pixels (~330 μm) was defined. Within each ROI, the presence of a dual-lobed DH-PSF was verified based on its expected lobe geometry and signal symmetry. If a valid DH-PSF was found, the central position, intensity, inter-lobe angle, and inferred depth (via angle-to-depth calibration) of emitters were computed. The final volumetric image was rendered by assigning the intensity at the 3D coordinate of localized emitters across all scanning angles.

Unlike the multifocal illumination, widefield illumination provides no prior information about the positions of DH-PSFs. To accurately and efficiently identify dual-lobed DH-PSF emitters, we adopted a clustering-assisted matching strategy based on Lloyd’s k-means algorithm. For each image frame, all refined emitter localizations were partitioned into spatial clusters, with the number of clusters $K$ adaptively set as a function of total emitter count. This pre-clustering step significantly reduced the combinatorial pairing complexity from $O({N}^{2})$ to $O(K\cdot {N}_{k}^{2})$, where ${N}_{k}$ denotes the number of emitters in cluster $k$. By limiting pairing operations to local neighborhoods, this approach greatly improved computational speed while maintaining high matching fidelity. Within each cluster, candidate emitter pairs were screened based on a set of geometric and photometric constraints, including inter-lobe distance, intensity similarity, and angular orientation. Only emitter pairs that satisfied these criteria and were consistent with the expected DH-PSF geometry were retained for depth estimation. This spatially constrained clustering approach preserved reconstruction precision while reducing spurious pairings, and demonstrated improved computational scalability compared to brute-force strategies. The resulting per-frame sparse depth maps were accumulated across time, and a robust per-pixel statistic was applied to suppress outliers and enhance reconstruction fidelity. A pseudocode summary of the main DH-PSF image reconstruction workflow is provided in the Supplementary Information (Supplementary Algorithm 1).

The reconstruction workflow was executed in MATLAB 2023 on a regular computer (Intel i9-13900H CPU, 16.0 GB RAM). Computational times are detailed in Supplementary Table 4.

### ADMM-based 3D reconstruction with DH-PSFs

Let ${{{\bf{x}}}}\,{\in }\,{{\mathbb{R}}}^{{N}_{x}\times {N}_{y}\times {N}_{z}}$ denote the unknown 3D emitter distribution and ${{{\bf{y}}}}\,{\in }{{\mathbb{R}}}^{{N}_{x}\times {N}_{y}}$ the recorded DH-encoded image. The forward model is

5 $${{{\bf{y}}}}=H{{{\bf{x}}}}+{{{\boldsymbol{n}}}}={\sum }_{z}{h}_{z}*{x}_{z}+{{{\boldsymbol{n}}}},$$

where ${h}_{z}$ denotes the experimentally calibrated DH-PSF at depth $z$, ${x}_{z}$ is the emitter distribution in the corresponding axial plane, $*$ denotes 2D convolution, and ${{\bf{n}}}$ represents measurement noise. To reconstruct ${{{\bf{x}}}}$, we solve the following regularized least-squares problem:

6 $${\min }_{{{{\bf{x}}}}\ge 0}\frac{1}{2}{{{\Vert }}H{{{\bf{x}}}}-{{{\bf{y}}}}{{\Vert }}}_{2}^{2}+\lambda {{{\Vert }}{{{\bf{x}}}}{{\Vert }}}_{1}+{\lambda }_{{TV}}{{{\Vert }}\nabla {{{\bf{x}}}}{{\Vert }}}_{1}.$$

Here, the ${l}_{1}$ term promotes sparsity of single-molecule emitters across the 3D volume, while the total variation (TV) term suppresses noise and enforces local spatial consistency. The optimization is carried out with the ADMM^64^, which alternates between a quadratic update of the volume ${{\bf{x}}}$ and soft-thresholding steps for the sparsity and TV terms (Supplementary Note 2). Compared with brute-force or clustering-based approaches, the ADMM formulation offers a key advantage: it can robustly resolve heavily overlapping DH PSFs even under high emitter densities, as validated using phantoms consisting of fluorescent beads at different densities (Supplementary Fig. 5).

### Segmentation, vessel graph extraction and A/V labeling

Blood vessels were segmented by first accumulating all tracks persisting for at least three frames, followed by Gaussian blurring and Otsu thresholding of the resulting volume. The segmentation was refined using morphological closing.

A vessel graph was extracted by skeletonizing the segmented volume using the Voreen library’s blood vessel-optimized algorithm, with the bulge-size parameter heuristically optimized to 1^65^. In the resulting graph, nodes represent vessel branching points, and edges correspond to vessel segments between them. Flow direction for each edge was estimated by averaging local flow orientations along the edge’s skeleton voxels, using the local vessel radius as a spatial constraint.

Automated artery/vein labeling was performed using a recently described global graph optimization method. Briefly, the algorithm leverages the vessel graph topology and known flow directions. Given the high reliability of tracked flow directions, the option to flip flow direction of an edge was disabled. Incorrectly fused A/V crossing points, which can occur when arteries and veins are closely spaced in depth, were resolved by optimizing connectivity to minimize normalized mass flow discrepancies at branch points. Arteries and veins were then classified based on their characteristic diverging (arteries) and converging (veins) flow patterns.

### RBC labeling and characterization

RBCs for fluorescence labeling were isolated from donor littermate mice under anesthesia. Fluorescence labeling was performed using a commercial membrane labeling dye called DiD (1,1’-Dioctadecyl-3,3,3’,3’-Tetramethylindodicarbocyanine, D7757, ThermoFisher Scientific, USA), following a previously established protocol^66^. Briefly, the collected blood sample was diluted 10-fold with Dulbecco’s phosphate-buffered saline (DPBS, D8537, Sigma-Aldrich, USA) and centrifuged at 60 × *g* for 8 min at room temperature to remove plasma and buffy coat. This washing step was repeated three times to obtain purified RBCs. The resulting RBC pellet was resuspended in 640 µL of DPBS and mixed with 640 µL Diluent C (CGLDIL, Sigma-Aldrich, USA), along with 3.2 µL of DiD dye (50 mg/mL), and incubated for 30 seconds. Unbound dye was removed by four rounds of centrifugation at 60 × *g* for 8 min, with DPBS washes between cycles. The DiD-stained RBCs were inspected using a commercial microscope (Cytation C10, BioTek, USA), where the majority of stained RBCs retained their characteristic biconcave disk morphology (Supplementary Fig. 11a). ~2 × 10⁶ DiD-labeled RBCs were injected into each mouse before imaging. Assuming a total blood volume of 1.5 mL for a 20 g mouse and an average RBC density of ~8.13 × 10^6^ cells/µL^67,68^, the labeled RBCs account for only ~0.017% of the total RBC population. Additionally, we quantified the number of detectable labeled RBCs over ~25-min observation window (Supplementary Fig. 11b). Linear regression fit revealed a slow decrease trend (clearance rate: -43.41 cells/s), suggesting no acute destabilization or rapid clearance of the labeled RBCs under the experimental conditions.

### Animal experiments

All animal experiments were conducted in accordance with the Swiss Federal Act on Animal Protection and approved by the Cantonal Veterinary Office Zurich (ZH182/2023) and by the Animal Experimentation Ethics Committee of Tongji University (TJTJ01925102), Shanghai.

#### Animal model

C57BL/6 J mice (*N* = 6, 9–14 weeks old, female, Charles River Laboratories, USA) were used to evaluate imaging performance of both systems, with three mice assigned to each configuration. Athymic Foxn1nu mice (*N* = 3, 13–14 weeks old, female, Charles River Laboratories, USA) were used for tumor studies. Mice were housed in individually ventilated, temperature-controlled cages at 21–23 °C and 40–60% relative humidity under a 12-h light/dark cycle. Pelleted food (3437PXL15, CARGILL) and water were provided ad libitum. Sex was not included as a variable in the experimental design or statistical analysis.

#### Surgery and anesthesia

Inhalation anesthesia was used with isoflurane (5% for induction, 1.5% for maintenance) with a gas mixture of oxygen (0.1 L/min) and air (0.4 L/min). During surgery and imaging, the mouse was immobilized using a stereotaxic frame (Narishige International Limited, UK), and body temperature was maintained at 37 °C with a feedback-controlled heating system (PhysioSuite, Kent Scientific, USA). Analgesia was provided via subcutaneous injection of buprenorphine (0.1 mg/kg) 30 min prior to scalp removal and craniotomy. Laser-scanning imaging was performed transcranially after scalp removal, whereas widefield imaging was conducted through a cranial window. This window was created via a 3 × 3 mm^2^ craniotomy over the primary somatosensory cortex using a dental drill and sealed with a glass coverslip. Prior to imaging, a catheter filled with phosphate-buffered saline (PBS) was inserted into the tail vein for intravenous injection. Under multifocal illumination configuration, 50 µL of 2 mg/mL fluorescent dye Cy5.5 was administered, whereas for widefield experiments, 100 µL of stained RBC suspension (~2 × 10^7^ cells/mL) was injected. After the imaging experiment, the mice were euthanized under deep isoflurane anesthesia (5%) for 5 minutes followed by cervical dislocation.

#### Intracranial tumor implantation

The U87-MG human glioblastoma cell line (Catalog Nr: 300367, CLS Cell Lines Service GmbH, Germany) was cultured in Eagle’s Minimum Essential Medium (EMEM, Sigma Aldrich) with 10% FBS (FBS, Sigma Aldrich) in a constant temperature incubator set at 37 °C abounding with 5% CO₂. Cells were trypsinized, washed, and resuspended in PBS at 1 × 10⁷ cells/mL for following orthotopic tumor transplantation. Briefly, nude mice were anesthetized with isoflurane (5% for induction and 1.5–2% for maintenance) and received buprenorphine (0.1 mg/kg, s.c.) 30 min before surgery. After scalp disinfection, a ~ 1 cm midline incision was made, and a 0.5-mm burr hole was drilled 1.0 mm posterior to bregma and 1.7–2 mm lateral. A 2 µL suspension (1 × 10⁶ cells) was injected stereotactically into the brain (0.6–1.2 mm depth) over 15–20 min. The hole was sealed with bone wax, and the scalp was sutured. Postoperative care followed institutional guidelines. In this study, orthotopic implantation achieved a 100% survival rate and resulted in tumor growth in all mice. Out of 5 injected mice, we selected 3 mice with tumors growing in the cortex (Supplementary Fig. 8) and excluded the other two because their tumors were situated in the hippocampus. This high level of reproducibility is supported by a standardized cell-culture protocol, stereotactic implantation performed by experienced operators, and precise anatomical targeting guided by a mouse brain atlas. Routine magnetic resonance imaging (MRI) monitoring of tumor development further contributed to the success rate by validating successful engraftment and ensuring that all animals reached an adequate tumor size for imaging analyses. Animals were monitored regularly and euthanized at humane endpoints, including tumor volume ≥1200 mm³ or earlier upon signs of distress (e.g., ulceration, neurological impairment, pain, or >10% body-weight loss). The approved tumor burden limit was not exceeded. Although inter-animal variability in tumor growth rates was observed, longitudinal MRI scans enabled accurate scheduling of downstream experimental procedures at biologically appropriate time points.

#### Magnetic resonance imaging

Tumor development and anatomical localization were monitored using high-resolution MRI. T1-weighted anatomical reference images were acquired using a two-dimensional fast low angle shot (2D FLASH) sequence. 32 contiguous axial slices were obtained covering the brain from dorsal to ventral. Imaging parameters were as follows: flip angle = 30°, repetition time = 549.7 ms, echo time = 3.5 ms, bandwidth = 44,642.9 Hz, FOV = 15.6 × 15.218 mm^2^, matrix size = 312 × 312, and slice thickness = 0.25 mm, resulting in an effective spatial resolution of 0.050 × 0.049 × 0.25 mm^3^. MRI scans were used to verify tumor engraftment, determine tumor location relative to the imaged FOV in the cortex, and schedule subsequent functional imaging experiments.

### Reporting summary

Further information on research design is available in the Nature Portfolio Reporting Summary linked to this article.

## Supplementary information

Supplementary Information

Description of Additional Supplementary Files

Supplementary Movie 1

Supplementary Movie 2

Supplementary Movie 3

Supplementary Movie 4

Reporting Summary

Transparent Peer Review file

## Source data

Source Data

## Data Availability

The main data supporting the finding of this study are available within the main text or Supplementary Information. Representative raw imaging datasets supporting the findings of this study have been deposited in Zenodo and are publicly available at 10.5281/zenodo.18876905. Source data are provided with this paper.

## Code availability

Localization of fluorescence emitters was performed with the open-source TrackNTrace toolbox^63^. RBC tracking was performed using the SimpleTracker algorithm^36^. ADMM reconstruction was performed with the open-source DiffuserCam code^69^. Pseudocode outlining the main DH-PSF image reconstruction workflow is included in the Supplementary Information (Supplementary Algorithm 1). Custom MATLAB codes for data analysis which are available for research purposes from the corresponding author upon request.
