# Supplementary material for: Double-helix optical point spread function enables real-time mesoscopic 3D functional microangiography in the living mouse brain and skull
Source: Nat Commun. 2026 Apr 13;17:5167. doi: 10.1038/s41467-026-71746-9 (PMC13249855; doi:10.1038/s41467-026-71746-9)
Supplement: Supplementary file 1 — Supplementary Information [file 41467_2026_71746_MOESM1_ESM.pdf]

Supplementary Information for

Double-helix optical point spread function enables real-time mesoscopic 3D functional microangiography in the living mouse brain and skull

Baoyuan Zhang[1,2,3], Shiyao Guo[4], Lin Tang[1,2], Yi Chen[1,2], Lukas Glandorf[1,2], Etienne Jessen[5], Xuyang Chang[1,2], Tian Jin[1,2], Michael Reiss[1,2], Shuxin Lyu[1,2], Qiang Fu[6], Hadi Amata[6], Wolfgang Heidrich[6], Chaim Glück[1], Dominik Schillinger[5], Bruno Weber[1], Xosé Luís Deán-Ben[1,2], Weibo Wang[3,*], Xiong Dun[4,*], Daniel Razansky[1,2,*] , Zhenyue Chen[4,*], and Quanyu Zhou[1,2,*]

[1] Institute of Pharmacology and Toxicology, Faculty of Medicine, University of Zurich, Zurich, Switzerland.

[2] Institute for Biomedical Engineering, Department of Information Technology and Electrical Engineering, ETH Zurich, Zurich, Switzerland.

[3] Center of Ultra-Precision Optoelectronic Instrument Engineering, Harbin Institute of Technology, Harbin, China.

[4] School of Physics Science and Engineering, Tongji University, Shanghai, China.

[5] Institute for Mechanics, Computational Mechanics Group, Technical University of Darmstadt, Darmstadt, Germany.

[6] King Abdullah University of Science and Technology, Thuwal, Saudi Arabia.

*Correspondence: wwbhit@hit.edu.cn, dunx@tongji.edu.cn, daniel.razansky@uzh.ch, zhenyue_chen@tongji.edu.cn, quanyu.zhou@uzh.ch.

Supplementary Note 1	Quantitative analysis of DH-PSF depth estimation robustness and precision
Supplementary Note 2	Resolving overlapping emitters in depth using ADMM
Supplementary Figure 1	Systematic analysis of factors influencing DH-PSF depth estimation precision
Supplementary Figure 2	Impact of phase mask manufacturing errors on the maximum intensity of DH-PSF across different depths
Supplementary Figure 3	Comparison of PSF engineering strategies: DH phase mask versus cylindrical lens
Supplementary Figure 4	Resolving axially overlapping emitters using ADMM-based DH-PSF reconstruction
Supplementary Figure 5	ADMM-based reconstruction in phantoms containing different densities of fluorescent emitters
Supplementary Figure 6	ADMM-based reconstruction in phantoms with fluorescent emitters under different scattering conditions
Supplementary Figure 7	Differentiation of microvessels using axial flow dynamics and depth traces
Supplementary Figure 8	T1-weighted anatomical MRI images acquired using 2D FLASH
Supplementary Figure 9	Tumor-induced shift in the distribution of vessel trajectory tortuosity
Supplementary Figure 10	Iterative phase optimization framework for depth-coded DH-PSF generation

Supplementary Figure 11	Assessment of DiD-stained RBC integrity and stability
Supplementary Table 1	Performance comparison between the proposed method and other state-of-the-art mesoscopic imaging techniques
Supplementary Table 2	Design parameters of DH phase masks
Supplementary Table 3	Step-by-step fabrication procedures of DH phase mask
Supplementary Table 4	Computational time of the reconstruction workflow
Supplementary Algorithm 1	DH-PSF image reconstruction workflow

Other supporting materials for this manuscript include:

Supplementary Video 1: Dynamic visualization of cerebral-to-calvarial vascular perfusion

Supplementary Video 2: 3D vascular architecture

Supplementary Video 3: Trajectory-resolved RBC flow in selected vascular region

Supplementary Video 4: Counter-propagating RBC flow in tumor vasculature

Supplementary Note 1: Quantitative analysis of DH-PSF depth estimation robustness and precision

This note presents a detailed analysis quantifying the robustness of the DH-PSF depth estimation accuracy (σ_z) under variations in photon count and noise level. To achieve this, we performed a comprehensive three-phase Monte-Carlo simulation study designed to generate high-quality statistical data (Supplementary Fig. 1).

Phase 1: Robustness to photon count (N)

We evaluated the system's performance by sweeping N (500, 1000, 2000, 2000, 5000, 10000) across five different axial positions ranging from 0 µm to 800 µm. The depth precision (σ_z) across all tested z levels decreases monotonically and significantly with N, from 9.19 µm at $N = 500$ to 0.93 µm at $N = 10000$.

Phase 2: Robustness to camera read noise (σ_{read})

We specifically evaluated the sensitivity to camera read noise using a fixed photon count (N = 2000) and a fixed depth. The depth precision σ_z remains essentially stable for modest read noise levels (σ_{read} = 1-5 ADU), fluctuating only between 2.68 µm and 3.08 µm. Precision degrades more noticeably only at high read noise (σ_{read} = 10 ADU to $\sigma_z \approx$ 3.88 µm).

Phase 3: Robustness to background noise (B_0)

We assessed robustness to uniform background noise by varying B_0 (N = 2000, fixed depth). We observed that modest backgrounds (B_0 = 10–50 ADU) mildly increase σ_z (≈2.10–2.50 µm), whereas large background levels (B_0 = 200–500 ADU) substantially degrade performance ($\sigma_z \approx$ 4.28 µm and 6.44 µm, respectively).

Our results confirm that the DH-PSF method maintains high accuracy and scales its precision primarily according to theoretical limits, with background noise identified as the dominant practical limiting factor.

Supplementary Note 2: Resolving overlapping emitters in depth using ADMM

Resolving spatially overlapping emitters remains a major challenge in 3D single-molecule localization microscopy, particularly under high-density conditions where conventional algorithms, assuming isolated PSFs, fail to recover emitter positions accurately. While existing PSF engineering strategies (e.g., astigmatic, biplane, and tetrapod designs) expand axial range, they remain vulnerable to PSF overlap. In contrast, the DH-PSF encodes axial information as lobe rotation, allowing inherent disambiguation of emitters with PSF overlapping. We addressed the inverse localization problem by implementing the ADMM-based reconstruction framework, enabling accurate 3D reconstruction from spatially overlapping DH-encoded images. We validated the reconstruction method using two phantom experiments performed under both laser scanning microscopy and widefield microscopy equipped with DH-PSF sensing modules.

The first phantom experiment was designed to create two fluorescent targets with the same xy location but different z positions and to test whether the ADMM method could distinguish them. The phantom was generated using Cy5.5 dye immobilized on two coverslips, with axial separation introduced by stacking blank glass slides. DH-encoded images were acquired under multifocal illumination. As shown in Supplementary Fig. 4b, increasing axial offset yielded partially overlapping PSFs with distinct rotational signatures, which were accurately separated in the reconstructed 3D volumes (middle and bottom rows). Supplementary Fig. 4c presents a representative image containing regions with single- and double-layer fluorescence. In the corresponding reconstruction, single-emitter regions were localized to one axial plane, while overlapping regions resolved into two discrete z positions (Supplementary Fig. 4d).

The second phantom experiment was designed to examine whether the ADMM method can resolve more general PSF overlapping cases, where emitters are closely spaced both laterally and axially, and to evaluate the fidelity of ADMM reconstruction under different densities of fluorescent emitters and scattering conditions. Fluorescent particles (~10 μm diameter, 540/560, F8833, Thermo Fisher, USA) were immobilized in 0.65% agar to create a random distribution of fluorescent emitters in a 3D volume. Intralipid (20%, MedchemExpress, USA) was suspended in the agar to mimic tissue scattering. DH-encoded images were acquired under widefield illumination. For validation, the same phantom was imaged with a widefield microscope equipped with a 0.25-NA objective, followed by z-scan to render the 3D volume.

To examine the influence of fluorescent emitter density on ADMM performance, fluorescent particles at densities of 7.2×10^3/ml, 7.2×10^4/ml, and 3.6×10^5/ml were imaged (Supplementary Fig. 5). As particle density increased, more overlapping PSFs were observed. ADMM accurately recovered emitter locations at densities of 7.2×10^3/ml and 7.2×10^4/ml. At the highest density (3.6×10^5/ml), ADMM still reconstructed all bead positions; however, some incorrect localizations were observed (5/33 beads), especially in regions of PSF overlap. In high-density scenarios, the reconstruction degrades because the underlying signal is no longer strictly sparse. This mismatch violates the sparsity prior, causing the regularization term to lose its efficacy in suppressing artifacts.

We further investigated the influence of sample scattering properties on ADMM performance (Supplementary Fig. 6). Intralipid at different concentrations (0%, 0.6%, 1.2%) was used to mimic the average optical scattering in biological tissues[1], corresponding to reduced scattering coefficient μ_s' of 0, 8.64, and 17.28 cm^{-1}, respectively. In the absence of scattering (0%) or under low scattering (0.6%), ADMM reconstruction correctly recovered all bead localizations. Under increased scattering (1.2%), ADMM still detected all beads, although a small number of incorrect reconstructions were observed (2/9 beads). The reconstruction errors are expected to arise from two aspects: (1) the increasing scattering causes the practical PSF to deviate from the ideal PSF used for reconstruction, and (2) stronger scattering decreases the SNR of the acquired images.

Collectively, these results confirm the method's ability to resolve spatially and axially overlapping emitters over a practical range of emitter densities and scattering properties.

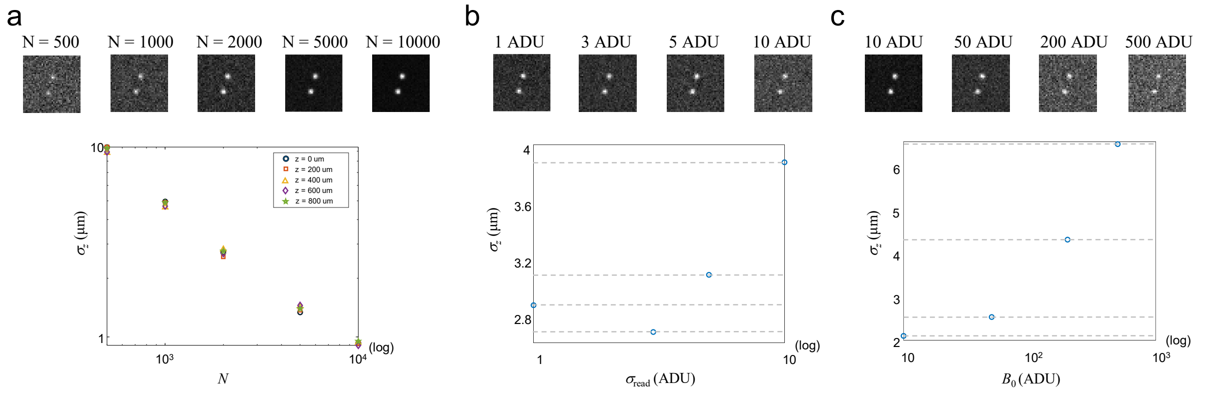

Supplementary Figure 1. Systematic analysis of factors influencing DH-PSF depth estimation precision. a-c Dependence of estimation precision (σ_z) on photon count (N), camera readout noise (σ_{read}), and background noise (B_0). Source data of Supplementary Fig. 1 are provided as a Source Data file.

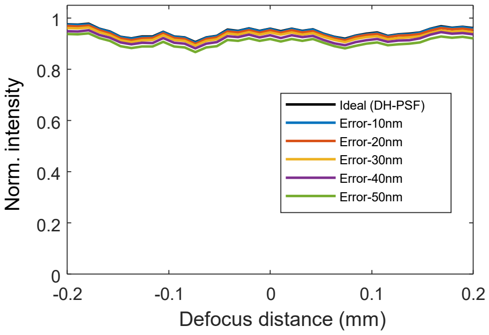

Supplementary Figure 2. Impact of phase mask manufacturing errors on the maximum intensity of DH-PSF across different depths.

Source data of Supplementary Fig. 2 are provided as a Source Data file.

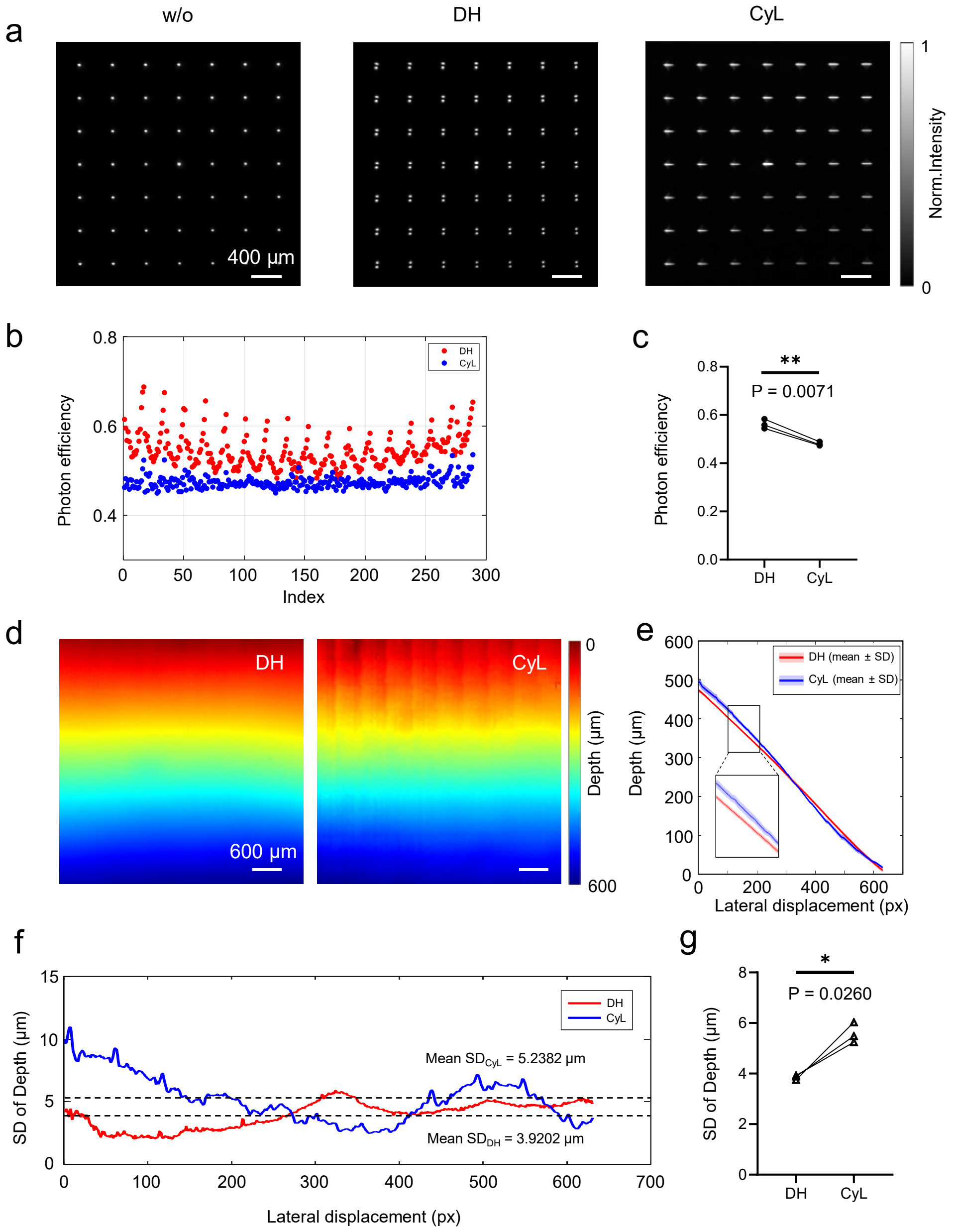

Supplementary Figure 3. Comparison of PSF engineering strategies: double-helix phase mask versus cylindrical lens. a Representative multifocal spot patterns under three PSF conditions: no phase modulation (left), double-helix PSF (middle), and cylindrical lens (right). Representative image from n = 3 independent experiments, with similar results observed across experiments. **b** Relative transmission efficiency across all 17×17 illumination spots, normalized to the maximum intensity in the no-mask condition. **c** Averaged transmission photon efficiency for DH and CyL across 17×17 grid positions. For each experiment, photon efficiency was computed across all 289 illumination spots and averaged to obtain one value per condition (n = 3 independent experiments). Statistical significance was assessed using a two-sided paired t-test ($P = 0.0071$; $t(2) = 11.77$). **d** Schematic of depth calibration using a tilted fluorescent slide. **e** Comparison of depth estimation profiles based on lateral displacement. **f** Standard deviation of depth localization, showing improved axial precision with DH-PSF. **g** Comparison of the SD of depth estimation for DH-PSF and CyL (n = 3 independent experiments). Statistical significance was assessed using a two-sided paired t-test (3.85 µm vs. 5.58 µm; $P = 0.0260$; $t(2) = 6.081$). $^{*}P < 0.05$; $^{**}P < 0.01$. Source data of Supplementary Fig. 3b, c, e, f and g are provided as a Source Data file.

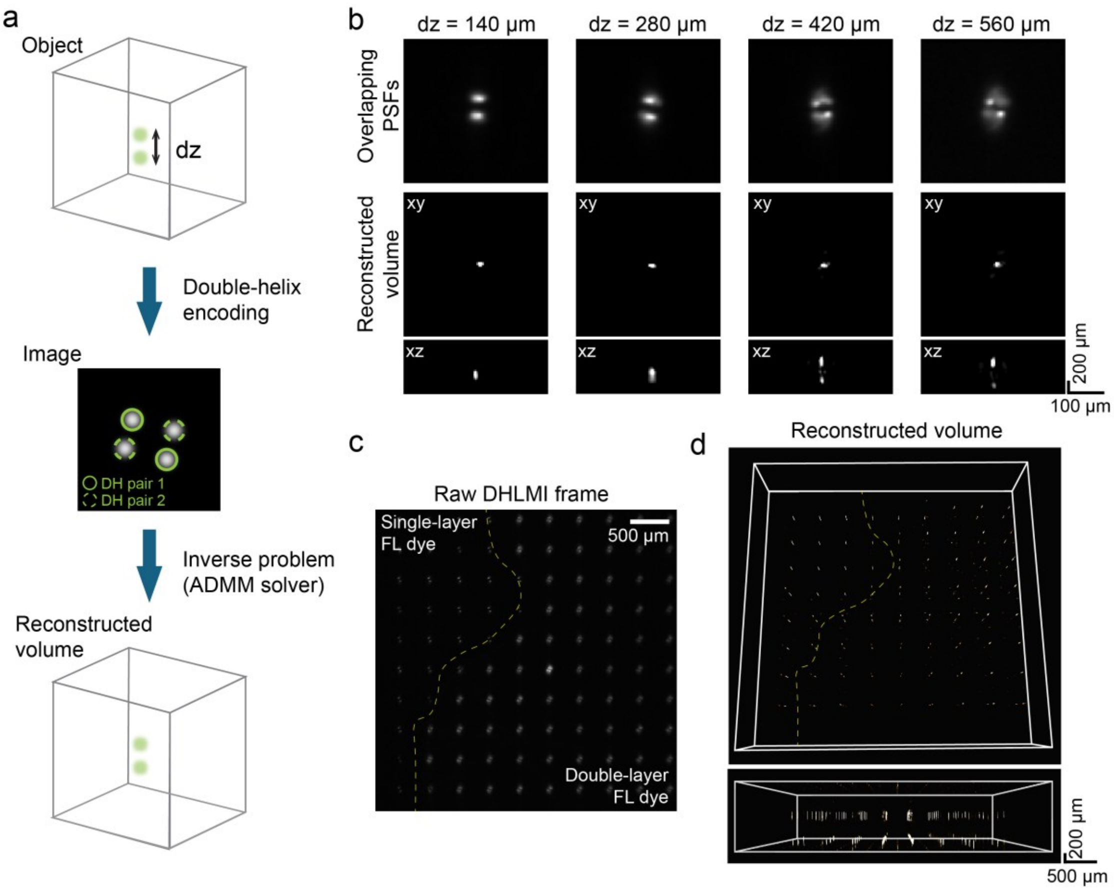

Supplementary Figure 4. Resolving axially overlapping emitters using ADMM-based DH-PSF reconstruction. a Schematic illustration of ADMM-based reconstruction under DH-PSF overlap conditions. **b** PSF patterns from two emitters located at the same lateral position but separated in depth, under varying axial distances. The corresponding reconstructed volumes are shown as maximum intensity projections in the xy and xz planes. **c** Representative raw DH-encoded image acquired in the phantom experiment. The dashed line indicates the boundary between single-layer and double-layer coverslip regions. **d** Reconstructed 3D volume showing spatial separation of emitters in the axial direction.

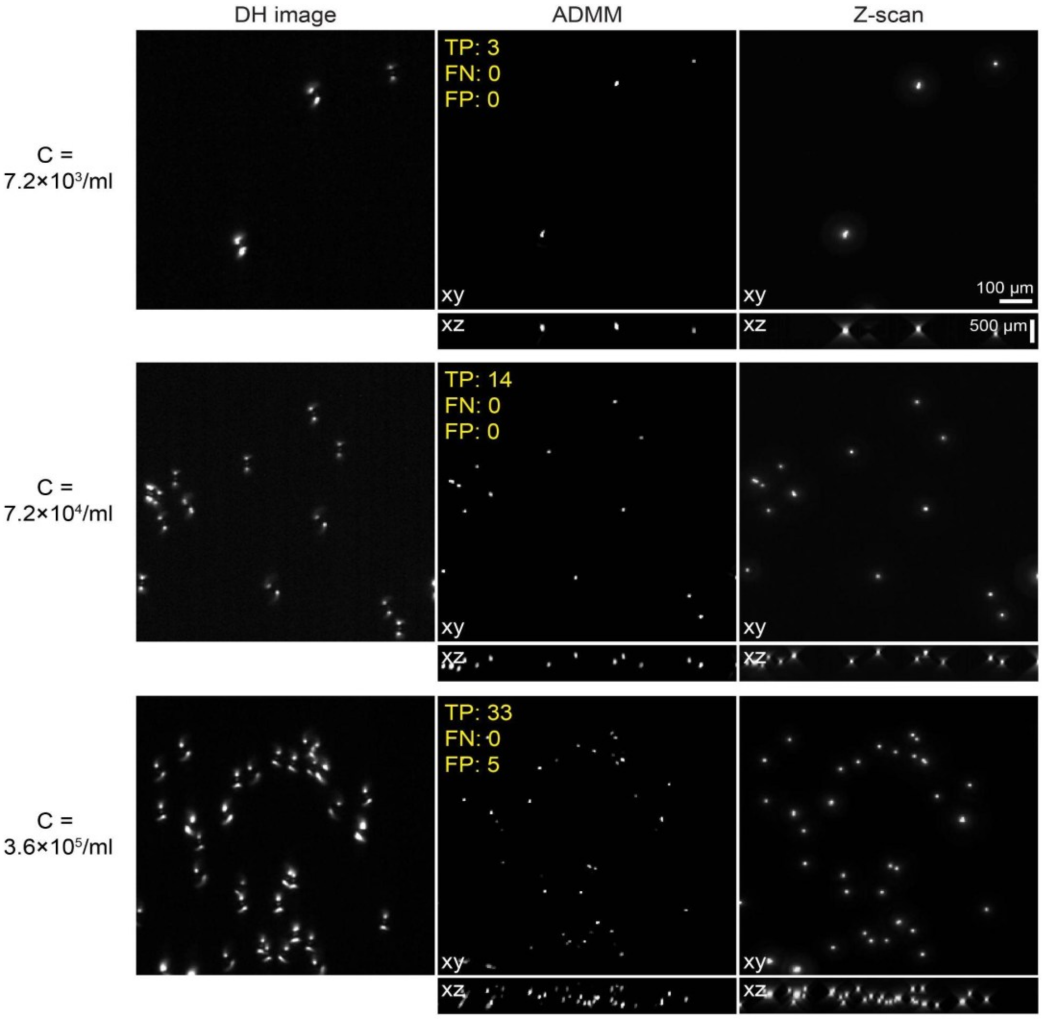

Supplementary Figure 5. ADMM-based reconstruction in phantoms containing different densities of fluorescent emitters. Fluorescent beads at three densities (7.2×10^3/ml, 7.2×10^4/ml, 3.6×10^5/ml) were embedded in 0.65% agar and imaged using widefield microscopy equipped with the DH-PSF sensing module. The same phantoms were imaged using widefield microscopy with a 0.25-NA objective and z-scan, which serves as the reference. Left: raw image acquired with DH-PSF sensing module. Middle: xy- and xz-plane projections following ADMM-based reconstruction. Right: xy- and xz-plane projections of the 3D volume acquired with the 0.25-NA objective and z-scan. By comparing the ADMM-reconstructed images with the reference images, true positive (TP), false negative (FN), and false positive (FP) bead detections were identified and labeled in the images.

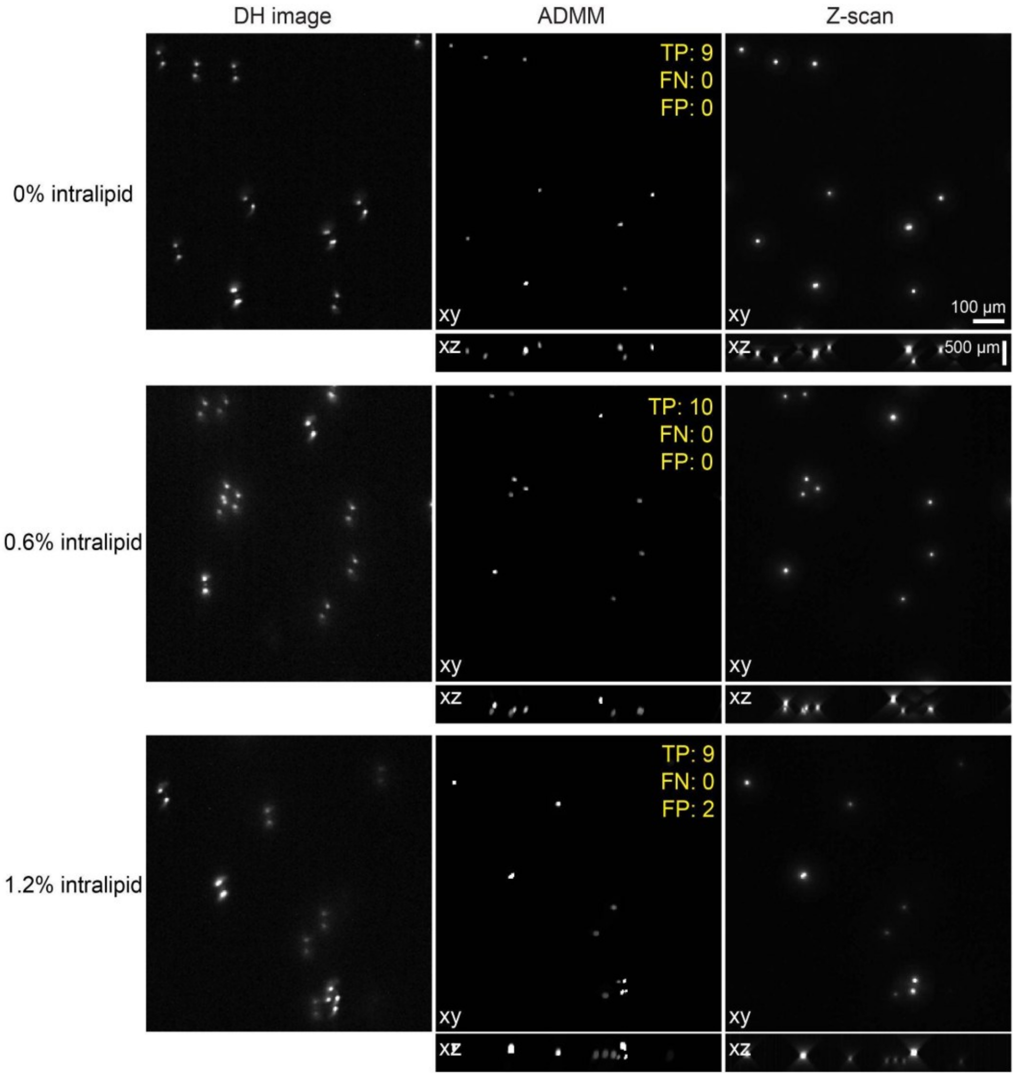

Supplementary Figure 6. ADMM-based reconstruction in phantoms with fluorescent emitters under different scattering conditions. Fluorescent beads at a density of 7.2×10^4/ml were embedded in agar/intralipid phantoms with varying intralipid concentrations (0% intralipid, 0.6% intralipid, 1.2% intralipid) to mimic different scattering conditions. The same phantoms were imaged using widefield microscopy with a 0.25-NA objective and z-scan, which serves as the reference. Left: raw image acquired with DH-PSF sensing module. Middle: xy- and xz-plane projections following ADMM-based reconstruction. Right: xy- and xz-plane projections of the 3D volume acquired with the 0.25-NA objective and z-scan. By comparing the ADMM-reconstructed images with the reference images, true positive (TP), false negative (FN), and false positive (FP) bead detections were identified and labeled in the images.

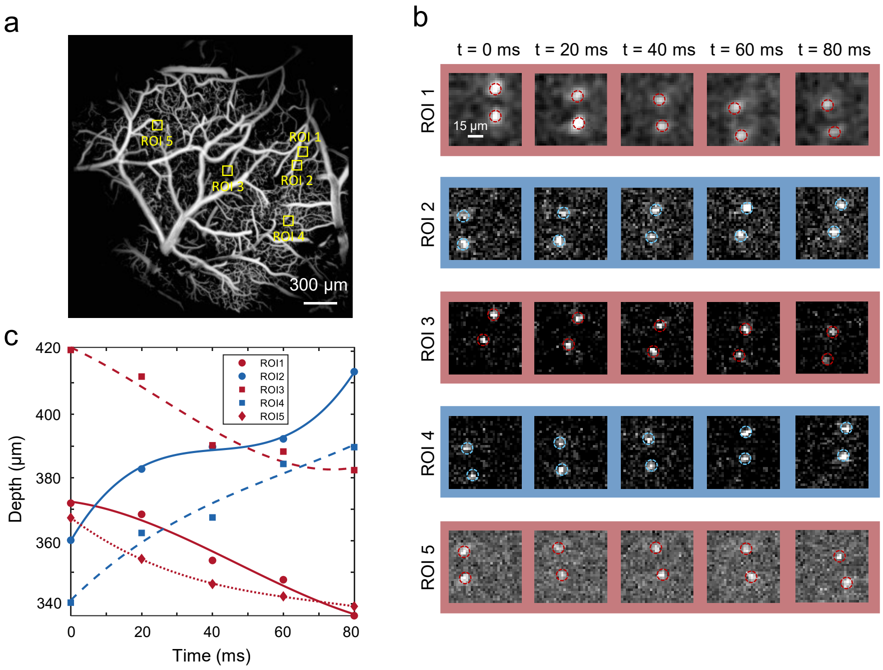

Supplementary Figure 7. Differentiation of microvessels using axial flow dynamics and depth traces. a Location of five representative regions of interest (ROI 1–ROI 5) on the intensity map. **b** Time-lapse DH-PSF images from the five selected ROIs. **c** Time-depth traces for all five ROIs (ROI1-ROI5), plotted with a third-order polynomial fit. Source data of Supplementary Fig. 7c are provided as a Source Data file.

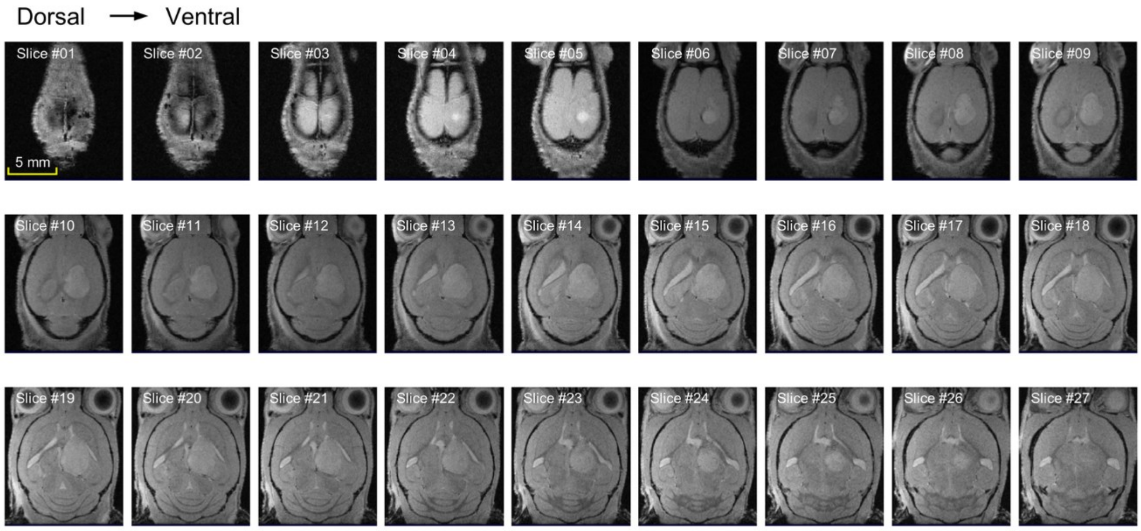

Supplementary Figure 8. T1-weighted anatomical MRI images acquired using 2D FLASH. For anatomical reference, a 2D FLASH sequence was used to acquire 32 contiguous axial slices covering the brain from dorsal to ventral.

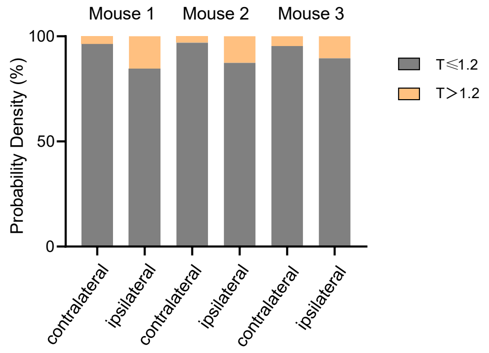

Supplementary Figure 9. Tumor-induced shift in the distribution of vessel trajectory tortuosity. Quantification of vessel trajectory tortuosity (T) distribution across individual mice (n = 3 mice, vessel trajectories analyzed across all mice). This 100% stacked bar chart presents the percentage distribution of vessel trajectory tortuosity in the contralateral and ipsilateral regions. The gray sections ($T \leq 1.2$) represent less tortuous vessels, and the orange sections ($T > 1.2$) represent highly tortuous vessels. The graph demonstrates that the proportion of highly tortuous trajectories ($T > 1.2$) is visibly and substantially increased in the ipsilateral side across all mice. Source data of Supplementary Fig. 9 are provided as a Source Data file.

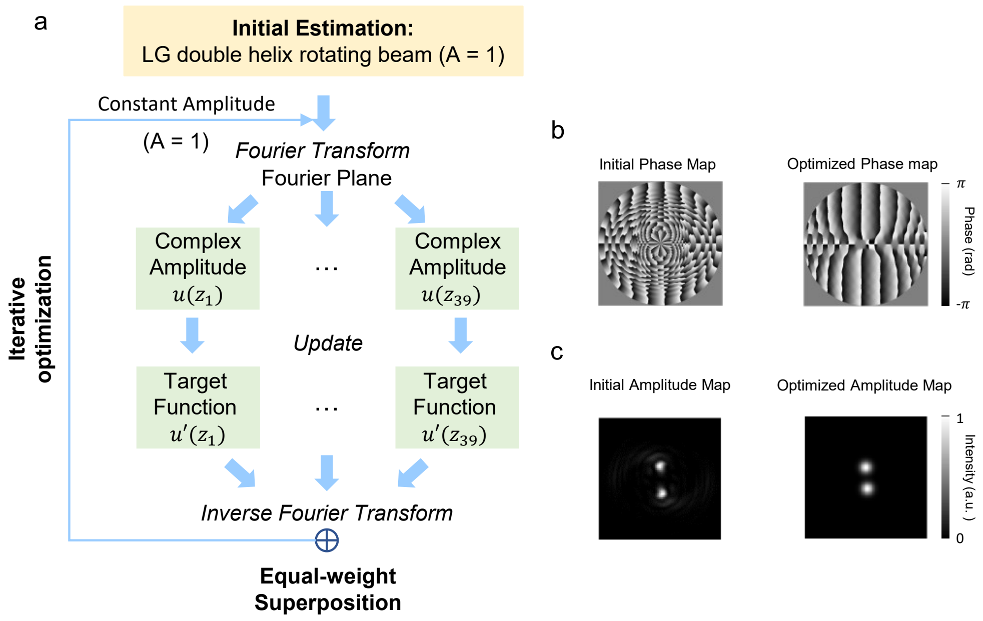

Supplementary Figure 10. Iterative phase optimization framework for depth-encoded double-helix PSF generation. a Schematic overview of the phase mask optimization workflow. **b** Comparison of the initial and optimized phase maps. **c** Corresponding amplitude maps derived from the initial and optimized phase masks.

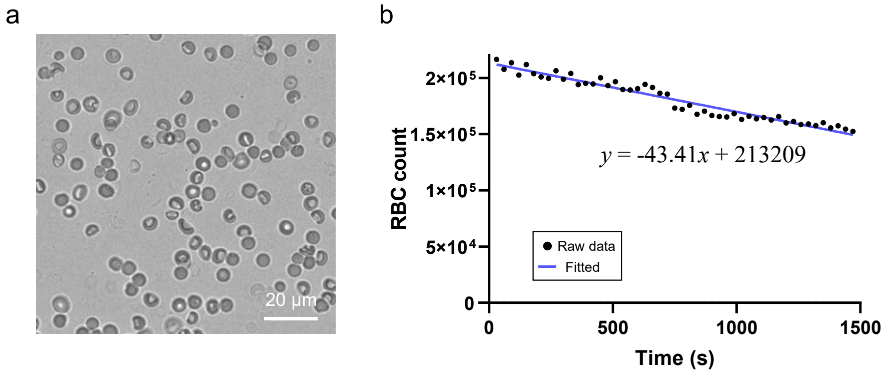

Supplementary Figure 11. Assessment of DiD-stained RBC integrity and stability. a Representative brightfield image of DiD-stained RBCs confirming the retention of the biconcave disk morphology following the staining protocol. Representative image from $n = 3$ independent experiments, with similar results observed across experiments. **b** The RBC count was plotted against time (s) over a 25-min period and fitted with a simple linear regression (blue line) to determine the leakage rate. The fitting result indicates a slow and stable decrease in cell count over the measurement period. Source data of Supplementary Fig. 11b are provided as a Source Data file.

Modality	Penetration Depth	Field of view	Lateral resolution	Axial resolution	Temporal Resolution	Surgical procedures	References
DH mesoscopy (laser scanning mode)	~300 µm with intact skull	6.6×6.6 mm^2	8.61 µm	3.92 µm depth estimation precision	3 Hz (3D)	Transcranial	This work
2PM	820 µm	500×425 μm^2	0.68-1.46 µm	10 µm	10 Hz (2D)	Cranial window	[2]
2PM with AO	400 µm	96×96 μm^2	near-diffraction-limited	2-3 µm	AO: ~1 min 1.5-3 Hz (2D)	Craniotomy	[3]
	850 µm	100×100 μm^2	near-diffraction-limited	>2 µm	——	Cranial window/ Skull thinning	[4]
3PM with AO	1.4 mm	40×40 μm^2	near-diffraction-limited	2–6 µm	9-36 s (AO + 3D)	Craniotomy	[5]
OAM	200–300µm	3×2 mm^2	3 µm	15 µm	1 Hz (3D)	Transcranial	[6]
OCT	2.3 mm	3×3 mm^2	15 µm	5-10 µm	0.2 Hz (3D，assuming 400 B-scans)	Cranial window	[7]

Supplementary Table 1: Comparison of system performance between the proposed method and other state-of-the-art mesoscopic imaging techniques. Abbreviations: DH, Double-helix; 2PM, two-photon microscopy; 3PM, three-photon microscopy; AO, adaptive optics; OAM, optoacoustic microscopy; OCT, optical coherence tomography.

**Depth range**	**Angle range**	**Aperture size**	**LG mode**	**σ**	**T_{TL} (Tube lens)**	**Beam waist radius ω_0**
[-0.4, 0.4] mm	180°	18 mm	(*l*,*p*)=(9,0) (11,1) (13,2) (15,3) (17,4) (19,5) (21,6) (23,7) (25,8) (27,9) (29,10)	2	105 mm, 200 mm	1.6 mm (aperture size/11)
[-0.4, 0.4] mm	180°	18 mm	(*l*,*p*)=(1,0) (3,2) (5,4) (7,6) (9,8) (11,10)	2	200 mm	1.7 mm (aperture size/10.5)
[-0.4, 0.4] mm	100°	18 mm	(*l*,*p*)=(9,3) (11,6) (13,9) (15,12) (17,15) (19,18) (21,21) (23,24)	2	105 mm, 200 mm	1.6 mm (aperture size/17.5)

Supplementary Table 2: Parameters of designed masks.

Step	Work	Tool	Process
1	Wafer cleaning	Wet bench	Rinse the wafer in Piranha solution (H_2SO_4 3:1 H_2O_2) for 10 min at 115 °C, followed by drying in a wafer drier for 7 min.
2	Cr deposition	Sputtering (ESCRD4)	Deposit 150 nm Cr thin film on the wafer for 9 min 15 s.
3	Adhesion promotion	HMDS vapor prime (YES-310TA)	Apply adhesion promotion film for 20 min at 115 °C.
4	Spin coating	Spin coater	Spin coat 0.6 μm AZ1505 at 3,000 rpm.
5	Soft bake	Hot plate	Place the wafer on the hot plate for 1 min at 100 °C.
6	UV exposure	Mask aligner (EVG 6200)	Align the wafer with the mask on the contact aligner and expose the wafer with UV light with a dose of 9 mJ/cm^2.
7	Development	Wet bench	Rinse the wafer in AZ726MIF for 18 s.
8	Cr etching	Wet bench	Rinse the wafer in Cr Etchant (TechniEtch Cr01) for 2 min.
9	Sonication	Wet bench	Place the wafer in acetone for ultrasonic bath for 5 min.
10	Reactive-ion etching	RIE (Plasmalab 100 - ICP 380)	Etch the wafer with CHF_3 (15 sccm) and O_2 (5 sccm) at 10 °C.
11	Cr removal	Wet bench	Rinse the wafer in Cr Etchant (TechniEtch Cr01) for 5 min.
12	Repeat Steps #1–#11 for multi-level structures.		

Supplementary Table 3: Step-by-step fabrication procedures of DH phase masks.

Imaging system	Task	Time (s)	Notes
Laser scanning	Searching for the illumination spots	97	For one compound image, reconstructed from 400 raw frames (0.33 s recording time)
	Pairing and reconstruction	26	
Widefield	Localization and tracking	302	For 2371 raw frames (11.86 s recording time)
	Pairing and reconstruction	75	
	Flow velocity/direction calculation	52	

Supplementary Table 4: Computational time of the reconstruction workflow.

Supplementary Algorithm 1. DH-PSF image reconstruction workflow

Input: Raw image sequences I_n; candidate spot localizations L_n; calibration parameters $k(x,y)$, $b(x,y)$; frame rate f_s, pixel size s.
Output: Depth stack D.

Procedure:

Initialize Depth stack D.

For each image file $n = 1, \ldots, N$ **do**

Load image sequences I_n and candidate spot localizations L_n.

For each frame t **do**

Extract candidate spots $P_t=\{(x_i,y_i,I_i)\}_{i=1}^{M_t}$.

If $M_t<2$ **then continue.**

Partition P_t into K local groups by k-means: $P_t=\bigcup_{m=1}^{K} C_m$.

For each cluster C_m **do**

Identify valid pairs (p_i, p_j) satisfying

$$d_{min}<d_{ij}<d_{max},$$
$$|I_i-I_j|\leq\tau_I,$$
$$\theta_{min}<\theta_{ij}<\theta_{max}.$$

For each valid pair (p_i, p_j) **do**

Compute midpoint $(x_c,y_c)=\frac{1}{2}\left((x_i,y_i)+(x_j,y_j)\right)$.

Compute DH angle $\theta_{ij}=\text{atan2}\left(-(y_j-y_i),(x_j-x_i)\right)$.

Recover depth using local calibration $z_c=\frac{\theta_{ij}-b(x_c,y_c)}{k(x_c,y_c)}$.

Update current depth map $D_n^{(t)}(x_c,y_c)\leftarrow z_c$.

End for

End for

Update file-wise projection $D_n\leftarrow P_{max}\left(D_n,D_n^{(t)}\right)$.

End for

Append D_n to D.

End for

Return D.

References

1 Flock, S. T., Jacques, S. L., Wilson, B. C., Star, W. M. & van Gemert, M. J. C. Optical properties of intralipid: A phantom medium for light propagation studies. *Lasers in Surgery and Medicine* **12**, 510-519 (1992).

2 Wu, R. *et al.* A versatile miniature two-photon microscope enabling multicolor deep-brain imaging. *Nature Methods* **22**, 1935-1943 (2025).

3 Ji, N., Milkie, D. E. & Betzig, E. Adaptive optics via pupil segmentation for high-resolution imaging in biological tissues. *Nature Methods* **7**, 141-147 (2010).

4 Liu, R., Li, Z., Marvin, J. S. & Kleinfeld, D. Direct wavefront sensing enables functional imaging of infragranular axons and spines. *Nature Methods* **16**, 615-618 (2019).

5 Wang, T. *et al.* Three-photon imaging of mouse brain structure and function through the intact skull. *Nature Methods* **15**, 789-792 (2018).

6 Yao, J. *et al.* High-speed label-free functional photoacoustic microscopy of mouse brain in action. *Nature Methods* **12**, 407-410 (2015).

7 Choi, W. J. & Wang, R. Swept-source optical coherence tomography powered by a 1.3-μm vertical cavity surface emitting laser enables 2.3-mm-deep brain imaging in mice in vivo. *Journal of Biomedical Optics* **20**, 106004 (2015).
