# Supplementary material for: Double-helix optical point spread function enables real-time mesoscopic 3D functional microangiography in the living mouse brain and skull
Source: Nat Commun. 2026 Apr 13;17:5167. doi: 10.1038/s41467-026-71746-9 (PMC13249855; doi:10.1038/s41467-026-71746-9)
Supplement: Supplementary file 2 — Description of Additional Supplementary Files [file 41467_2026_71746_MOESM2_ESM.pdf]

Description of Additional Supplementary Files

Supplementary Movie 1: Dynamic visualization of cerebral-to-calvarial vascular perfusion.

Supplementary Movie 2: 3D vascular architecture.

Supplementary Movie 3: Trajectory-resolved RBC flow in a selected vascular region.

Supplementary Movie 4: Counter-propagating RBC flow in tumor vasculature.
