# Supplementary material for: Double-helix optical point spread function enables real-time mesoscopic 3D functional microangiography in the living mouse brain and skull
Source: Nat Commun. 2026 Apr 13;17:5167. doi: 10.1038/s41467-026-71746-9 (PMC13249855; doi:10.1038/s41467-026-71746-9)
Supplement: Supplementary file 7 — Reporting Summary [file 41467_2026_71746_MOESM7_ESM.pdf]

nature portfolio

Corresponding author(s): Weibo Wang, Xiong Dun, Zhenyue Chen, Daniel Razansky

Last updated by author(s): Mar 4, 2026

Reporting Summary

Nature Portfolio wishes to improve the reproducibility of the work that we publish. This form provides structure for consistency and transparency in reporting. For further information on Nature Portfolio policies, see our Editorial Policies and the Editorial Policy Checklist.

Statistics

For all statistical analyses, confirm that the following items are present in the figure legend, table legend, main text, or Methods section.

n/a	Confirmed	
☐	☒	The exact sample size (*n*) for each experimental group/condition, given as a discrete number and unit of measurement
☐	☒	A statement on whether measurements were taken from distinct samples or whether the same sample was measured repeatedly
☐	☒	The statistical test(s) used AND whether they are one- or two-sided<br>*Only common tests should be described solely by name; describe more complex techniques in the Methods section.*
☒	☐	A description of all covariates tested
☒	☐	A description of any assumptions or corrections, such as tests of normality and adjustment for multiple comparisons
☐	☒	A full description of the statistical parameters including central tendency (e.g. means) or other basic estimates (e.g. regression coefficient) AND variation (e.g. standard deviation) or associated estimates of uncertainty (e.g. confidence intervals)
☐	☒	For null hypothesis testing, the test statistic (e.g. *F*, *t*, *r*) with confidence intervals, effect sizes, degrees of freedom and *P* value noted<br>*Give P values as exact values whenever suitable.*
☒	☐	For Bayesian analysis, information on the choice of priors and Markov chain Monte Carlo settings
☒	☐	For hierarchical and complex designs, identification of the appropriate level for tests and full reporting of outcomes
☒	☐	Estimates of effect sizes (e.g. Cohen's *d*, Pearson's *r*), indicating how they were calculated

Our web collection on statistics for biologists contains articles on many of the points above.

Software and code

Policy information about availability of computer code

Data collection

1. Camware (version 4.12, PCO AG, Germany) was used for data collection both in laser scanning mode and wide-field mode.
2. ParaVision software (verision 6.0.1, Bruker BioSpin, Germany) was used for MRI data acquisition to monitor tumour development and anatomical localization in mice.

Data analysis

1. TrackNTrace (version 1.03, https://github.com/scstein/TrackNTrace, 2016) was used for localization and tracking.
2. The "vessel diameter.im" plugin (version 1.0) in ImageJ (version 1.54d, National institutes of Health, USA) was used to quantify the vessel diameter.
3. Custom MATLAB (R2023b, MathWorks, USA) codes were used for image reconstruction.
4. The "simpletracker" function (version 1.5.1, https://ww2.mathworks.cn/matlabcentral/fileexchange/34040-simpletracker) with MATLAB (R2023b, MathWorks, USA) was employed to 3D red blood cell tracking.
5. ADMM reconstruction was performed with the open-source DiffuserCam code (https://github.com/Waller-Lab/DiffuserCam).

For manuscripts utilizing custom algorithms or software that are central to the research but not yet described in published literature, software must be made available to editors and reviewers. We strongly encourage code deposition in a community repository (e.g. GitHub). See the Nature Portfolio guidelines for submitting code & software for further information.

Data

Policy information about availability of data

All manuscripts must include a data availability statement. This statement should provide the following information, where applicable:

- Accession codes, unique identifiers, or web links for publicly available datasets
- A description of any restrictions on data availability
- For clinical datasets or third party data, please ensure that the statement adheres to our policy

The main data supporting the finding of this study are available within the main text or Supplementary Information. Source data are provided with this paper. Representative raw imaging datasets supporting the findings of this study have been deposited in Zenodo and are publicly available at https://doi.org/10.5281/zenodo.18876905.

Research involving human participants, their data, or biological material

Policy information about studies with human participants or human data. See also policy information about sex, gender (identity/presentation), and sexual orientation and race, ethnicity and racism.

Reporting on sex and gender	Not applicable
Reporting on race, ethnicity, or other socially relevant groupings	Not applicable
Population characteristics	Not applicable
Recruitment	Not applicable
Ethics oversight	Not applicable

Note that full information on the approval of the study protocol must also be provided in the manuscript.

Field-specific reporting

Please select the one below that is the best fit for your research. If you are not sure, read the appropriate sections before making your selection.

☒ Life sciences ☐ Behavioural & social sciences ☐ Ecological, evolutionary & environmental sciences

For a reference copy of the document with all sections, see nature.com/documents/nr-reporting-summary-flat.pdf

Life sciences study design

All studies must disclose on these points even when the disclosure is negative.

Sample size	No statistical methods were used to predetermine sample size. Sample sizes were chosen based on previous studies using similar in vivo imaging approaches and practical considerations associated with animal experiments. For transcranial laser-scanning imaging (Fig. 2), n = 3 mice were used. For widefield cerebrovascular imaging (Figs. 3–4), n = 3 mice were used. For glioma imaging (Figs. 5–6), n = 3 mice with cortical tumors were analyzed. Exact sample sizes for each experiment are stated in the figure legends and/or main text.
Data exclusions	Two mice were excluded from glioma imaging analyses because tumors were located in the hippocampus rather than the cortex. No other data were excluded.
Replication	Transcranial imaging (Fig. 2), widefield cerebrovascular imaging (Figs. 3–4), and glioma imaging (Figs. 5–6) were each performed in n = 3 mice, with consistent results observed across animals.
Randomization	Randomization was not performed because this study did not include randomized group comparisons and primarily reports a proof-of-concept imaging method.
Blinding	Blinding was not performed because no randomized group comparisons were conducted in this proof-of-concept study.

Reporting for specific materials, systems and methods

We require information from authors about some types of materials, experimental systems and methods used in many studies. Here, indicate whether each material, system or method listed is relevant to your study. If you are not sure if a list item applies to your research, read the appropriate section before selecting a response.

Materials & experimental systems

n/a	Involved in the study
☒	☐ Antibodies
☐	☒ Eukaryotic cell lines
☒	☐ Palaeontology and archaeology
☐	☒ Animals and other organisms
☒	☐ Clinical data
☒	☐ Dual use research of concern
☒	☐ Plants

Methods

n/a	Involved in the study
☒	☐ ChIP-seq
☒	☐ Flow cytometry
☐	☒ MRI-based neuroimaging

Eukaryotic cell lines

Policy information about cell lines and Sex and Gender in Research

Cell line source(s)	The human glioblastoma cell line U87-MG (Catalog No. 300367) was obtained from CLS Cell Lines Service GmbH (Germany).
Authentication	The cell line was used as provided by the supplier and was not further authenticated in our laboratory.
Mycoplasma contamination	The cell line tested negative for mycoplasma contamination.
Commonly misidentified lines (See ICLAC register)	U87-MG is listed in the ICLAC register of commonly misidentified cell lines. The cell line was used as a widely established glioblastoma model in accordance with previous studies.

Animals and other research organisms

Policy information about studies involving animals; ARRIVE guidelines recommended for reporting animal research, and Sex and Gender in Research

Laboratory animals	C57BL/6J mice (N = 6, 9 – 14 weeks old, female, Charles River Laboratories, Germany) were used to evaluate imaging performance of both systems, with three mice assigned to each configuration. Athymic Foxn1nu mice (N = 3, 13 – 14 weeks old, female, Charles River Laboratories, USA) were used for tumor studies.
Wild animals	No wild animals were used in the study.
Reporting on sex	All n = 9 mice utilized across the experiments (Figures 2–6) were female, as reflected in the overall animal numbers reported. A sex-based analysis was not performed because the primary objective of this study was to demonstrate and validate the technical concept of a novel mesoscopic cerebrovascular structural and functional imaging methodology. The design was focused on methodological proof-of-concept and technical performance rather than assessing biological variability, which rendered sex and gender differences irrelevant to the core findings.
Field-collected samples	No field-collected samples were used in the study.
Ethics oversight	All animal experiments were conducted in accordance with the Swiss Federal Act on Animal Protection and approved by the Cantonal Veterinary Office Zurich (ZH182/2023) and by the Animal Experimentation Ethics Committee of Tongji University (TJTJ01925102), Shanghai.

Note that full information on the approval of the study protocol must also be provided in the manuscript.

Plants

Seed stocks	Not applicable
Novel plant genotypes	Not applicable
Authentication	Not applicable

Magnetic resonance imaging

Experimental design

Design type	MRI was used only for anatomical reference for tumour localization.
Design specifications	The mouse was anesthetized with isoflurane and placed in the MRI scanner. After acquisition of T1-FLASH anatomical images, the animal was removed from the scanner.
Behavioral performance measures	Behavioral performance was not evaluated in this study, as the mice were euthanized after the experiments before awakening from anesthesia.

Acquisition

Imaging type(s)	Anatomical images.
Field strength	7 T.
Sequence & imaging parameters	T1-weighted anatomical reference images were acquired using a two-dimensional fast low angle shot (2D FLASH) sequence. 32 contiguous axial slices were obtained covering the brain from dorsal to ventral. Imaging parameters were as follows: flip angle = 30°, repetition time = 549.7 ms, echo time = 3.5 ms, bandwidth = 44,642.9 Hz, FOV = 15.6×15.218 mm2, matrix size = 312×312, and slice thickness = 0.25 mm, resulting in an effective spatial resolution of 0.050×0.049×0.25 mm3.
Area of acquisition	Anatomical MRI was primarily performed on mouse brain regions containing tumours; therefore, deeper brain regions without tumours were not included in the imaging field.

Diffusion MRI ☐ Used ☒ Not used

Preprocessing

Preprocessing software	ParaVision 6.0.1 was used as the user interface for exporting TIFF images of the mouse brain.
Normalization	Not applicable.
Normalization template	Not applicable.
Noise and artifact removal	Not applicable.
Volume censoring	Not applicable.

Statistical modeling & inference

Model type and settings	Not applicable.
Effect(s) tested	Not applicable.

Specify type of analysis: ☐ Whole brain ☐ ROI-based ☐ Both

Statistic type for inference (See Eklund et al. 2016)	Not applicable.
Correction	Not applicable.

Models & analysis

n/a	Involved in the study
☒	☐ Functional and/or effective connectivity
☒	☐ Graph analysis
☒	☐ Multivariate modeling or predictive analysis
