# Supplementary material for: Double-helix optical point spread function enables real-time mesoscopic 3D functional microangiography in the living mouse brain and skull
Source: Nat Commun. 2026 Apr 13;17:5167. doi: 10.1038/s41467-026-71746-9 (PMC13249855; doi:10.1038/s41467-026-71746-9)
Supplement: Supplementary file 8 — Transparent Peer Review file [file 41467_2026_71746_MOESM8_ESM.pdf]

nature portfolio

Peer Review File

Double-helix optical point spread function enables real-time mesoscopic 3D functional microangiography in the living mouse brain and skull

Corresponding Author: Professor Daniel Razansky

This file contains all reviewer reports in order by version, followed by all author rebuttals in order by version.

Version 0:

Reviewer comments:

Reviewer #1

(Remarks to the Author)
This manuscript presents a mesoscopic fluorescence imaging platform based on a double-helix phase mask, enabling real-time depth-resolved measurements of cerebrovascular networks and microcirculation in the living mouse brain through an intact skull. The platform features a compact, generalizable design compatible with both laser scanning and widefield microscopy.

Key technical advancements include a spatially adaptive calibration strategy to correct field-dependent aberrations, a 3.25-fold improvement in photon efficiency over conventional Laguerre-Gaussian (LG)-based methods via optimized phase mask design, and an ADMM-based reconstruction approach to resolve overlapping point spread functions (PSFs).

Functionally, the system achieves a spatial resolution of 8.76 ×8.76 ×3.92 μm3 over a 6.6 ×6.6 ×0.8 mm3 volume (space-bandwidth product of 1.16 ×108) at a 3 Hz volume rate. It extracts hemodynamic parameters: perfusion time-to-peak (TTP) from laser scanning mode and flow velocity/direction from widefield tracking of fluorescently labeled red blood cells (RBCs).

In applications, the platform discriminates between calvarial and cerebral vasculature, maps brain-layer-specific perfusion dynamics, and characterizes tumor-associated vascular remodeling (e.g., increased tortuosity, disrupted continuity, bidirectional flow) in glioma-bearing mice, demonstrating utility in both physiological and pathological cerebrovascular studies.

Major Revision
The manuscript reports a valuable imaging technology with significant advancements in mesoscopic 3D cerebrovascular imaging. Its technical innovations, functional versatility, and in vivo validation in glioma models address critical limitations of existing modalities. However, several substantive issues (detailed below) need clarification and supplement to ensure the rigor, reproducibility, and comprehensiveness of the work before acceptance.

Specific Comments and Questions
Technical Details and Reproducibility
1.If this technology is to be widely applied, it is necessary to conduct a thorough assessment of its universality and potential application scope.Can this technology be used to view brain structures other than blood vessels and how to label them?
2. The phase mask fabrication process mentions using imprint lithography with AZ-1512 photoresist and PDMS replication, but key parameters (e.g., photoresist spin-coating speed, exposure dose, PDMS curing temperature ramp) are missing. Please provide these details to enable reproducibility.
3. For the spatially adaptive calibration strategy (17×17 grid partitioning), how was the grid size determined? Was a sensitivity analysis conducted to confirm that 17×17 is optimal (e.g., comparing with smaller/larger grid sizes on depth retrieval error)?
4. The ADMM-based reconstruction approach is introduced to resolve overlapping PSFs, but the manuscript only references Supplementary Note 2 and Fig. 3. Please add a brief description in the main text of the algorithm’s core constraints (e.g., sparsity terms, regularization parameters) and how it outperforms brute-force or other clustering methods.

Functional Validation and Quantification
1. When comparing DH-PSF with cylindrical lens-based PSF engineering (Supplementary Fig. 2), the manuscript reports a 48.3% improvement in photon transmission efficiency and lower depth estimation standard deviation (3.92 μm vs. 5.24 μm). However, it does not specify how many independent experiments were conducted for this comparison. Please provide the sample size (n) and statistical significance tests (e.g., t-test) to support these claims.
2. In the transcranial dye perfusion experiments (Fig. 2), brain vessels are reported to be 50–150 μm deeper and have 3–6 s earlier TTP than skull vessels. Are these differences consistent across all 6 C57BL/6J mice? Please present pooled data (e.g., box plots) of depth and TTP differences between brain and skull vessels across animals.
3. For flow velocity measurements in widefield mode (Fig. 3), the manuscript distinguishes between transverse (2D-projected) and total (3D) velocity. What is the average percentage difference between 2D and 3D velocity values across different vessel types (arterioles, capillaries, venules)? This data would highlight the necessity of 3D velocity mapping.

Limitations and Future Work
1. The manuscript notes that laser scanning mode underutilizes camera sensor area due to sparse excitation. It proposes wavelength multiplexing or polarization labeling as solutions, but no preliminary data (e.g., feasibility tests, expected improvement in sensor utilization) is provided. Please add a brief discussion of whether these strategies have been tested in pilot experiments.
2. Deep learning-based reconstruction is suggested as a potential alternative to k-means clustering. Are there any initial results (e.g., comparison of localization accuracy/speed between deep learning and current methods) that can be included to strengthen this proposal?
3. The system's effective penetration depth through the intact skull is ~300 μm. How does this compare to other transcranial imaging modalities (e.g., two-photon microscopy with adaptive optics, optoacoustic imaging)? A concise table in the Discussion section comparing penetration depth, FOV, and temporal resolution would contextualize the platform's performance.

Animal Experiments and Ethical Considerations
1. For the glioma model (U87-MG cell injection), the manuscript states that nude mice were used, but it does not specify the number of mice with successful tumor implantation (e.g., if 3 mice were injected, how many developed tumors suitable for imaging?). Please clarify the tumor implantation success rate and exclude any animals with no/low tumor burden from the analysis.
2. In the RBC labeling protocol, DiD dye is used at 50 mg/mL, and 3.2 μL is added to the RBC suspension. Is there any evidence of dye toxicity or impact on RBC viability/circulation time (e.g., comparing labeled vs. unlabeled RBC half-life in pilot studies)?
3. The manuscript mentions that mice were euthanized with ketamine overdose post-imaging. Please confirm that the ketamine dose complies with the Swiss Federal Act on Animal Protection (e.g., dose per gram of body weight) and provide this detail for transparency.

Typos and Language:

The manuscript is well-written, but a few minor corrections are suggested:
Page 2, Line 74: "depth-retrival uncertainty" should be "depth-retrieval uncertainty".
Page 5, Line 257: "This phenomenon has predominantly manifested" could be rephrased to "This phenomenon was predominantly observed" .

Reviewer #2

(Remarks to the Author)
In the manuscript the authors present a novel mesoscopic fluorescence imaging platform which is based on a double-helix phase mask finalized at performing high-speed volumetric imaging of cerebral and calvarial vasculature structures. Double-helix point spread function imaging has been already proposed in microscopy imaging, but here the authors very convincingly extend the technique into the mesoscopic area providing detailed guidance on techniques and strategies for concequent artifacts removal.

I think that the possibility to perform (as abundantly shown here) in-vivo depth-resolved large-area imaging at the capillary scale through the mouse intact skull is a game-changer of extremely great relevance and offers great potential for probing cerebrovascular alterations as here indicated. It could also find extended use in the cardiovascular field I would imagine.

The manuscript is extremely well written. A large amount of carefully given details is present, and it's very clear in its content, description, and purpose. I found both the exposition and the methodology very clear. Also the language is pretty fluent. The supplementary information is also extremely well done.

Data are very nicely collected and the images appear to be of great quality. I was greatly impressed by the amount of work the authors spent not only in the system characterization but also on how extensively they have tried to demonstrate its use in the biological imaging context.

As a minor thing I would explain better the classical double helix point spread function strategy in the introductory section in order to better set a basis for what comes next in the paper. Probably not everybody if familiar with it.

Regarding the figures they are all excellent in both quality and content description.
In Figure 1, in panel “b” it is a little bit difficult to see clearly what is going on.
Panel “h” indicates a later resolution of 8.76 microns. I would guess this is calculated on one fitted bead. It would be probably better to analyze on more RBCs?

Reviewer #3

(Remarks to the Author)
This manuscript presents a new approach to mesoscopic fluorescence imaging by introducing a high-efficiency double-helix point spread functon(DH-PSF) scheme for real-time, depth-resolved microangiography in the intact mouse brain. The work addresses a long-standing challenge in neuro-imaging, namely achieving rapid, volumetric visualization of large-scale cerebrovascular networks without compromising spatial resolution, field-of-view, or functional sensitivity.

Conventional two-photon and localization-based optical methods offer either high spatial resolution or functional flow measurements, but not both simultaneously at mesoscale dimensions. Ultrasound and optoacoustic localization methods provide volumetric access but at the expense of optical resolution, complexity, or transcranial performance. Point spread functon(PSF) engineering has emerged as a promising strategy to achieve depth-resolved optical imaging, where axial information is encoded into the PSF shape, however, particularly DH-PSF has previously not been successfully extended to mesoscopic imaging due to low transmission efficiency and severe field-dependent aberrations.

The authors overcome these limitations through a re-designed, 18-mm-aperture, DH phase mask optimised via iterative phase retrieval. Notably, it yields a 3.25-fold gain in photon efficiency. The authors then use a spatially adaptive calibration method to reduce depth-retrieval error by over 8x. When this scheme was combined with multifocal scanning, the system achieved real-time (3 Hz) volumetric imaging over a large 6.6×6.6×0.8 mm^3 field at ~9×9×4 μm^3 resolution. This enabled reliable discrimination between skull and cortical vessels and quantitative mapping of perfusion dynamics. In widefield mode, the platform further provides 3D flow velocity and direction measurements by tracking labeled RBCs.
The ability for both morphological and functional readouts makes the proposed method a powerful tool, which the authors applied to a few compelling use cases. The system was able to visualize depth-resolved perfusion patterns, reconstructed full cortical flow topology, and revealed marked structural and functional abnormalities in glioma vasculature, including increased density, tortuosity, and bidirectional flow.

Overall, this manuscript introduces a powerful and potentially widely applicable imaging tool which can provide a notable advance for high-throughput mesoscopic neurovascular imaging. As such the manuscript could be a good fit to Nature Communications, however, further clarification and additional validations, as outlined in more detail below, are suggested. Addressing these points would greatly enhance the clarity and impact of the work and bring it closer to the standards expected for a multidisciplinary journal.

Technical comments:
-) I suggest the authors include more systematic quantitative comparisons (resolution, accuracy, imaging depth, speeds, etc) against other state-of-the-art mesoscopic imaging methods, perhaps as a Supplementary Table?
-) How sensitive is DH-PSF performance to deviations in phase mask height profile or surface roughness? Has this been studied by the authors? Elaborating on this could be useful for further studies and for the general uptake of the technique.
-) Was photon efficiency measured across the entire FOV or only on-axis? How does transmission vary off-axis?
-) How robust is depth estimation to noise and variations in photon counts (signal intensity)?
-) A quantitative characterization of skull-induced aberrations (e.g., wavefront error maps) would significantly strengthen the claim of robust transcranial performance.
-) Regarding the ADMM-based reconstructions and resolution claims: Under high emitter density, how often does the ADMM approach mislocalize overlapped PSFs? How does this perform in more scattering phantoms? Phantom experiment mimicking more realistic conditions of the mouse brain could help to establish realistic error rates.
-) How sensitive is axial velocity estimation to depth noise, especially given the ~4 µm axial uncertainty?

Biological demonstrations and findings:
-) For the skull vs. brain vessel perfusion timing, is the observed separation consistent across all animals? Providing some statistics would strengthen the general findings, also for the other observations presented throughout Figs. 3-6.
-) Does the overall system performance differ significantly across animals? Has this been tested?
-) What is the minimal RBC labeling density required for stable flow reconstruction without excessive overlap?

Minor comments:
-) Could the authors provide more detail on computational requirements (processing time per volume) so one can assess the scalability for long recordings?

Version 1:

Reviewer comments:

Reviewer #1

(Remarks to the Author)

The authors have addressed most of my previous concerns through detailed explanations and substantial revisions to the manuscript. The additional data provided in the revised paper significantly strengthens the paper's conclusions.
However, a few minor points remain that require further clarification or technical refinement before the manuscript can be recommended for publication.

Data and Code Availability (Crucial for Reproducibility):
The manuscript relies heavily on custom algorithms (ADMM, K-means clustering) and a specialized phase plate design. To ensure the reproducibility of the findings and the utility of this platform for the broader imaging community, the authors should provide more than a "upon request" statement.
I suggest the authors deposit the core reconstruction scripts and a representative raw data sample (e.g., a short image sequence of the double-helix lobes) to a public repository like GitHub or Zenodo. At minimum, a detailed pseudo-code or a flowchart of the ADMM optimization process should be included in the Supplementary Information.

Reviewer #2

(Remarks to the Author)
The authors replie to all the comments I had. I also found the replies to the other reviewers' comments very insightful.

Reviewer #3

(Remarks to the Author)
I have read the revised manuscript by the authors and found it much improved. My comments and questions were thoroughly answered and addressed. In particular, the more quantitative comparison as well as characterisation of the system performance in the presence of noise add valuable details. The additional experiments statistics for the animal experiments also strengthen the findings of the manuscript. I therefore recommend publication.

Open Access This Peer Review File is licensed under a Creative Commons Attribution 4.0 International License, which permits use, sharing, adaptation, distribution and reproduction in any medium or format, as long as you give appropriate credit to the original author(s) and the source, provide a link to the Creative Commons license, and indicate if changes were made.
In cases where reviewers are anonymous, credit should be given to 'Anonymous Referee' and the source.
The images or other third party material in this Peer Review File are included in the article's Creative Commons license, unless indicated otherwise in a credit line to the material. If material is not included in the article's Creative Commons license and your intended use is not permitted by statutory regulation or exceeds the permitted use, you will need to obtain permission directly from the copyright holder.
To view a copy of this license, visit https://creativecommons.org/licenses/by/4.0/

Point-by-Point Response to the Reviewers' Comments

Reviewer #1

This manuscript presents a mesoscopic fluorescence imaging platform based on a double-helix phase mask, enabling real-time depth-resolved measurements of cerebrovascular networks and microcirculation in the living mouse brain through an intact skull. The platform features a compact, generalizable design compatible with both laser scanning and widefield microscopy.

Key technical advancements include a spatially adaptive calibration strategy to correct field-dependent aberrations, a 3.25-fold improvement in photon efficiency over conventional Laguerre-Gaussian (LG)-based methods via optimized phase mask design, and an ADMM-based reconstruction approach to resolve overlapping point spread functions (PSFs).

Functionally, the system achieves a spatial resolution of 8.76 ×8.76 ×3.92 μm^3 over a 6.6 ×6.6 ×0.8 mm^3 volume (space-bandwidth product of 1.16 $\times 10^8$) at a 3 Hz volume rate. It extracts hemodynamic parameters: perfusion time-to-peak (TTP) from laser scanning mode and flow velocity/direction from widefield tracking of fluorescently labeled red blood cells (RBCs).

In applications, the platform discriminates between calvarial and cerebral vasculature, maps brain-layer-specific perfusion dynamics, and characterizes tumor-associated vascular remodeling (e.g., increased tortuosity, disrupted continuity, bidirectional flow) in glioma-bearing mice, demonstrating utility in both physiological and pathological cerebrovascular studies.

Major Revision

The manuscript reports a valuable imaging technology with significant advancements in mesoscopic 3D cerebrovascular imaging. Its technical innovations, functional versatility, and in vivo validation in glioma models address critical limitations of existing modalities. However, several substantive issues (detailed below) need clarification and supplement to ensure the rigor, reproducibility, and comprehensiveness of the work before acceptance.

Reply: We thank the Reviewer for recognizing the value of our work and insightful suggestions. In the revised manuscript, we have carefully addressed the concerns with additional experiments, simulations, and analyses. Please find below our detailed responses to the specific comments.

Specific Comments and Questions

Technical Details and Reproducibility

1. If this technology is to be widely applied, it is necessary to conduct a thorough assessment of its universality and potential application scope. Can this technology be used to view brain structures other than blood vessels and how to label them?

Reply: The Reviewer raises an important aspect. While the current study focuses on vascular imaging, this choice was mainly motivated by the double-helix (DH) phase mask being optimized at a center wavelength of ~700 nm, which facilitates deeper *in vivo* penetration and signal-to-background ratio (SBR) with far-red fluorescent dyes. Since blood flow naturally provides sparse spatial features and vasculature can be readily labeled with fluorescent dyes, we selected the cerebrovascular network as our initial imaging target. However, the DH-based imaging system can potentially be applied to visualize other brain structures, i.e., neurons, astrocytes, and glial cells, provided that the phase mask is optimized for a specific excitation wavelength and suitable labeling strategies are implemented.

With respect to labeling approaches: (1) In widefield imaging scenarios, sparse fluorescent labeling is required to keep individual point emitters distinguishable from neighboring emitters. Such sparsity can be achieved through diluted viral transfections or cell type-specific Cre or CreER driver mouse lines [1]. (2) In laser-scanning imaging mode: sparse labeling is not strictly required, though it might be beneficial for enhancing image contrast. Thus, conventional genetically encoded fluorescent proteins [2] can be used to label cell populations more densely than in widefield mode, which greatly expands the flexibility of the proposed method. However, it's worth mentioning that fluorescent proteins with short emission wavelengths (e.g., ~510 nm emission peak for green fluorescent protein) are vulnerable to tissue scattering, whereas longer-wavelength variants tend to be dimmer, posing trade-offs in achievable resolution, penetration depth, and signal-to-noise ratio.

In the revised manuscript, a discussion on the applicability of the proposed method and the labeling strategies have been added to the *Discussion* section (page 7, line 333-342)

References:

[1] Veldman, Matthew B., et al. "Brainwide genetic sparse cell labeling to illuminate the morphology of neurons and glia with Cre-dependent MORF mice." Neuron 108.1 (2020): 111-127.

[2] Chan, Ken Y., et al. "Engineered AAVs for efficient noninvasive gene delivery to the central and peripheral nervous systems." Nature neuroscience 20.8 (2017): 1172-1179.

2. The phase mask fabrication process mentions using imprint lithography with AZ-1512 photoresist and PDMS replication, but key parameters (e.g., photoresist spin-coating speed, exposure dose, PDMS curing temperature ramp) are missing. Please provide these details to enable reproducibility.

Reply: We apologize for incorrect labeling of the fabrication method. The DH phase masks were fabricated using multi-level i-line photolithography and reactive-ion etching (RIE) on a fused silica wafer. We have revised the corresponding contents in the *Methods* section (page 9, line 435-442) to explicitly state this process flow and provide step-by-step fabrication procedure and parameters in the new Supplementary Table 4 for reproducibility.

Step	Work	Tool	Process
1	Wafer cleaning	Wet bench	Rinse the wafer in Piranha solution (H_2SO_4 3:1 H_2O_2) for 10 min at 115 ℃, followed by drying in a wafer drier for 7 min.
2	Cr deposition	Sputtering (ESCRD4)	Deposit 150 nm Cr thin film on the wafer for 9 min 15 s.
3	Adhesion promotion	HMDS vapor prime (YES-310TA)	Apply adhesion promotion film for 20 min at 115 ℃.
4	Spin coating	Spin coater	Spin coat 0.6 μm AZ1505 at 3,000 rpm.
5	Soft bake	Hot plate	Place the wafer on the hot plate for 1 min at 100 ℃.
6	UV exposure	Mask aligner (EVG 6200)	Align the wafer with the mask on the contact aligner and expose the wafer with UV light with a dose of 9 mJ/cm^2.
7	Development	Wet bench	Rinse the wafer in AZ726MIF for 18 s.
8	Cr etching	Wet bench	Rinse the wafer in Cr Etchant (TechniEtch Cr01) for 2 min.
9	Sonication	Wet bench	Place the wafer in acetone for ultrasonic bath

			for 5 min.
10	Reactive-ion etching	RIE (Plasmalab 100 - ICP 380)	Etch the wafer with CF_4 (15 sccm) and O_2 (5 sccm) at 10 °C.
11	Cr removal	Wet bench	Rinse the wafer in Cr Etchant (TechniEtch Cr01) for 5 min.
12	Repeat Steps #1-#11 for multi-level structures.		

Supplementary Table 4: Step-by-step fabrication procedures of DH phase masks.

3. For the spatially adaptive calibration strategy (17×17 grid partitioning), how was the grid size determined? Was a sensitivity analysis conducted to confirm that 17×17 is optimal (e.g., comparing with smaller/larger grid sizes on depth retrieval error)?

Reply: Selection of the 17×17 grid is based on (1) availability of beam-splitting gratings on the market, and (2) a tradeoff between depth fitting precision and SNR. Due to the limited commercially available options, we selected the off-the-shelf 17×17 grating rather than opting for a customized product. To address the second point, we performed an additional simulation, as detailed below.

The system's depth retrieval is based on the model $depth = (\theta\text{-}b)/k$, where depth is linearly linked to rotation angle of DH-PSF θ. The accuracy of k (slope) and b (intercept) are the critical calibration parameters that directly influence depth retrieval. We simulated the accuracy of k, b matrices under four grating configurations with different diffraction orders (N = 5×5, 11×11, 17×17, 29×29). Assuming a given k, b distribution as the ground truth, each grating corresponds to a different sampling density in the original k/b space. The resulting sampling positions on the k and b matrices are shown in the Response Fig. 1a, b. The final k and b maps were then obtained by bicubic interpolation based on these sampled points. Increasing N from 5 to 17 significantly reduces the root mean square error (RMSE) for both k_{RMSE} and b_{RMSE} (Response Fig. 1 c, top and middle panels). This confirms that a denser grid is crucial to accurately capture the non-uniform system aberrations and mitigate underfitting. However, increasing N reduces the illumination power deposited into each spot when the total energy is fixed (Response Fig. 1c bottom), thereby limiting the fluorescence signals in practical measurements. Moreover, high grid densities may increase the likelihood of PSF overlap. Taken together, the 17×17 configuration reflects a practical balance between maintaining adequate SNR per illumination spot and achieving sufficient spatial sampling to model field-dependent aberrations. We have added corresponding descriptions in the *Methods* section (page 8, line 391-393).

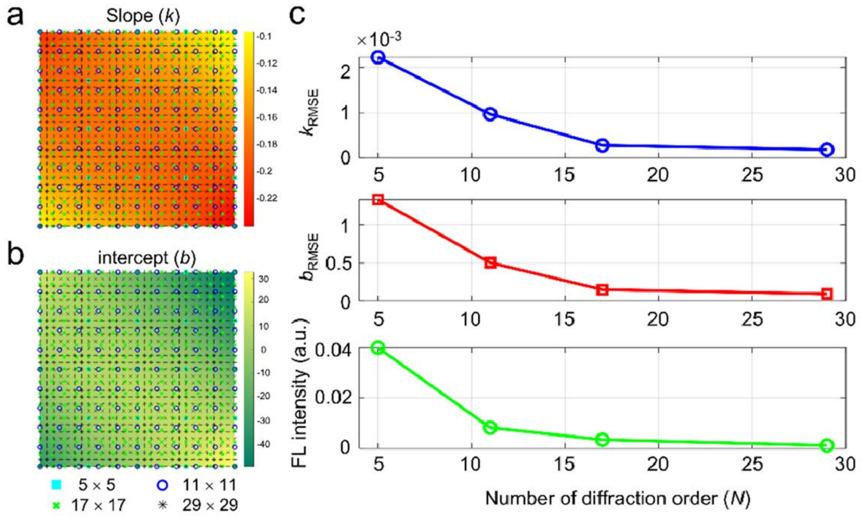

Response Figure 1. Simulations on the impact of the number of diffraction order (N) on calibration precision and fluorescence signal. FL: fluorescence.

4. The ADMM-based reconstruction approach is introduced to resolve overlapping PSFs, but the manuscript only references Supplementary Note 2 and Fig. 3. Please add a brief description in the main text of the algorithm's core constraints (e.g., sparsity terms, regularization parameters) and how it outperforms brute-force or other clustering methods.

Reply: As suggested, we have now added a detailed description of the ADMM-based reconstruction in the *Methods* section, detailing the sparsity and regularization terms (page 10, line 480-491). Compared with brute-force or clustering-based approaches, the ADMM formulation offers a key advantage: it can robustly resolve heavily overlapping DH PSFs even under high emitter densities, as validated in the newly added supplementary Fig. 5 using phantoms consisting of fluorescent beads with different densities. By contrast, brute-force or clustering methods typically succeed only when the dual lobes of the DH-PSF remain visually separable. The relevant discussion has been added to the *Methods* section (page 10, line 491-494).

Functional Validation and Quantification

1. When comparing DH-PSF with cylindrical lens-based PSF engineering (Supplementary Fig. 2), the manuscript reports a 48.3% improvement in photon transmission efficiency and lower depth estimation standard deviation (3.92 μm vs. 5.24 μm). However, it does not specify how many independent experiments were conducted for this comparison. Please provide the sample size (n) and statistical significance tests (e.g., t-test) to support these claims.

Reply: As suggested, we repeated the comparison between the DH- and cylindrical lens-based method in N = 3 independent measurements. To accurately capture the characteristic morphology of the PSF under double-helix phase mask and cylindrical lens, we employed a global analysis based on net integrated intensity, ensuring a robust estimation of photon throughput across all positions. In the revised Supplementary Fig. 3c, g, we have now included the statistical analyses on both metrics (transmission efficiency and standard deviation (SD) of depth estimation) and two-tailed paired t-tests. For photon transmission efficiency, the DH method yielded an average transmission efficiency of 0.54 compared to 0.47 for the cylindrical lens, with a P-value of 0.0071. For the SD of depth estimation, the DH method achieved an average value of 3.85 μm compared to 5.58 μm for the cylindrical lens, with a P-value of 0.0260. The corresponding descriptions have been added to the *Results* section (page 3, line 140-144) and Supplementary Fig. 3.

2. In the transcranial dye perfusion experiments (Fig. 2), brain vessels are reported to be 50–150 μm deeper and have 3–6 s earlier TTP than skull vessels. Are these differences consistent across all 6 C57BL/6J mice? Please present pooled data (e.g., box plots) of depth and TTP differences between brain and skull vessels across animals.

Reply: The transcranial dye perfusion experiment (Fig. 2) was repeated in N = 3 C57BL/6J mice. For statistical analysis, we selected n = 18 brain vessel and n = 18 skull vessels across 3 mice and presented the pooled depth and time-to-peak (TTP) data as box plots (new Fig. 2i, j). Statistical analysis using paired Student's t-tests confirmed significant disparities in both metrics. Brain vessels were found to be 94.40 ± 13.09 μm (mean ± SEM) deeper than skull vessels (P < 0.0001). Furthermore, brain vessels exhibited a significantly earlier TTP by 4.22 ± 0.53 s (P < 0.0001). The corresponding descriptions in the *Results* section have been updated (page 4, line 175-178).

3. For flow velocity measurements in widefield mode (Fig. 3), the manuscript distinguishes between transverse (2D-projected) and total (3D) velocity. What is the average percentage difference between 2D and 3D velocity values across different vessel types (arterioles, capillaries, venules)? This data would highlight the necessity of 3D velocity mapping.

Reply: Indeed, this is an important point. We have now quantified the systematic differences in absolute values between the transverse velocity (Vel_{2D}) and the total velocity (Vel_{3D}) across arteries, arterioles, capillaries, venules, veins, as shown in the Response Fig. 2a (which has been incorporated as the revised Fig. 3j in the manuscript). For analysis, n = 10 vessels per vessel type were selected from 3 mice.

The average percentage underestimation of the true velocity (calculated as (Vel_{3D} - Vel_{2D}) / Vel_{3D} × 100%) is presented in Response Fig. 2b. The underestimation is substantial and non-trivial, ranging from 24.62 ± 11.23 % in capillaries to 53.07 ± 13.91% in venules. The high underestimation in vessels, such as arteries (48.01 ± 10.36%) and venules, is attributed to the increased contribution of the axial velocity component, which is neglected in 2D projected measurements.

The corresponding descriptions have been added to the *Results* section (page 5, line 208-212) and Fig. 3.

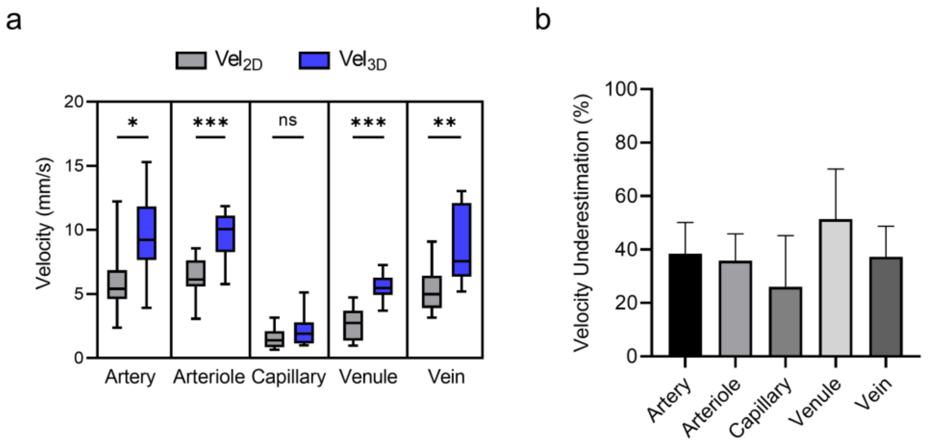

Response Figure 2. (a) Comparison of absolute Vel_{2D} (gray) and Vel_{3D} (blue) velocities, data presented as box plots. (n = 10 per vessel type, ns, not significant, *P < 0.05, **P < 0.01, ***P < 0.001). (b) Bar chart of percentage velocity underestimation across different vessel types.

Limitations and Future Work

1. The manuscript notes that laser scanning mode underutilizes camera sensor area due to sparse excitation. It proposes wavelength multiplexing or polarization labeling as solutions, but no preliminary data (e.g., feasibility tests, expected improvement in sensor utilization) is provided. Please add a brief discussion of whether these strategies have been tested in pilot experiments.

Reply: Wavelength multiplexing represents an important direction for future work. To evaluate its feasibility, we have conducted simulations where 488 nm and 660 nm lasers are combined from the illumination side with lateral displacement induced by diffraction grating. Due to the dispersion of beam-splitting grating, these two wavelengths would be projected at different positions on the sample plane, causing their corresponding DH-PSFs to occupy different pixel regions on the camera sensor (Response Fig. 3). This configuration effectively doubles the utilization efficiency of the camera sensor. Additional considerations, such as customized dual-band filters and the uniformity of illumination spots produced by the beam-splitting grating, will need to be addressed in practical implementations.

Polarization-based detection is not directly relevant for increasing the utilization rate of the camera. Although utilizing a polarization camera [1] or pupil-splitting methods [2] have been reported to exploit additional camera pixels and extract information such as the orientation of fluorescence emission dipole moments in super-resolution microscopy, these approaches are less informative in the mesoscopic regime. At this scale, molecular orientations are not fixed within the relatively large voxel volume, and therefore polarization-based methods may not

provide additional information. Thus, we revised the *Discussion* section to reflect this (page 7, line 347-350).

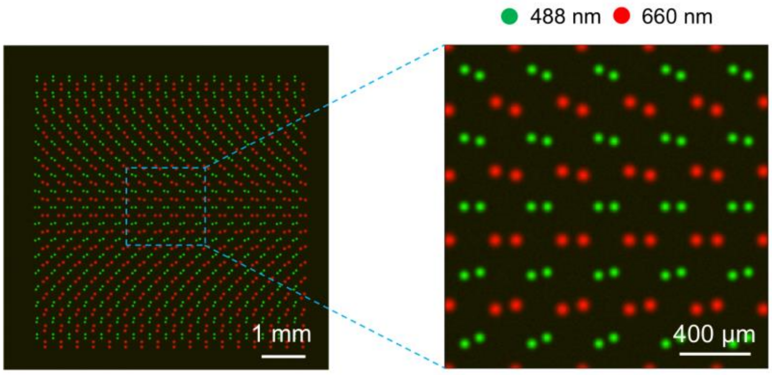

Response Figure 3. Simulated spatial intensity distribution under dual-wavelength excitation (λ_1 = 488 nm and λ_2 = 660 nm).

References:

[1] Bruggeman, Ezra, et al. "POLCAM: instant molecular orientation microscopy for the life sciences." Nature Methods 21.10 (2024): 1873-1883.

[2] Zhang, Oumeng, et al. "Six-dimensional single-molecule imaging with isotropic resolution using a multi-view reflector microscope." Nature photonics 17.2 (2023): 179-186.

2. Deep learning-based reconstruction is suggested as a potential alternative to k-means clustering. Are there any initial results (e.g., comparison of localization accuracy/speed between deep learning and current methods) that can be included to strengthen this proposal?

Reply: We appreciate the Reviewer's suggestion. At the current stage, we have not incorporated a deep-learning module into our pipeline nor performed comparisons between deep-learning methods and current k-means-based approach, mainly due to several practical challenges. In particular, we currently lack paired DH raw data and experimentally acquired ground-truth labels. A potential future direction is to generate synthetic training datasets with perfectly paired inputs and ground-truth outputs. However, several challenges must be addressed, such as accurately modeling depth- and wavelength-dependent PSF variations, asymmetric lobe morphology, and realistic noise levels. Without carefully considering these factors, it is difficult to assemble sufficiently representative training data, which may in turn compromise the generalizability of supervised models. We have added a detailed discussion of these factors in the revised manuscript and outlined our plan to generate realistic DH-PSF datasets to enable a fair evaluation in future work (page 7, line 355-359).

3. The system's effective penetration depth through the intact skull is ~300 μm. How does this compare to other transcranial imaging modalities (e.g., two-photon microscopy with adaptive optics, optoacoustic imaging)? A concise table in the Discussion section comparing penetration depth, FOV, and temporal resolution would contextualize the platform's performance.

Reply: We thank the Reviewer for the valuable suggestion. As suggested, we have added Supplementary Table 1 directly comparing the key performance parameters of the proposed system with state-of-the-art transcranial imaging modalities. We have referenced Supplementary Table 1 in the *Discussion* section to emphasize the advantages of our platform for multi-functional neuroimaging (page 7, line 322-324).

Animal Experiments and Ethical Considerations

1. For the glioma model (U87-MG cell injection), the manuscript states that nude mice were used, but it does not specify the number of mice with successful tumor implantation (e.g., if 3 mice were injected, how many developed tumors suitable for imaging?). Please clarify the tumor implantation success rate and exclude any animals with no/low tumor burden from the analysis.

Reply: In this study, orthotopic implantation achieved a 100% survival rate and resulted in tumor growth in all mice. Animals were selected based on tumor depth (3 of the 5 injected mice were included). We selected the mice with tumors located in the cortex (Supplementary Fig. 8) and excluded the other two because their tumors were situated in the hippocampus. This high level of reproducibility is supported by a standardized cell-culture protocol, stereotactic implantation performed by experienced operators, and precise anatomical targeting guided by a mouse brain atlas. Routine MRI monitoring of tumor development further contributed to the success rate by confirming successful engraftment and ensuring that all animals reached an adequate tumor size for imaging analyses. Although inter-animal variability in tumor growth rates was observed, longitudinal MRI scans enabled accurate scheduling of downstream experimental procedures at biologically appropriate time points. The corresponding descriptions have been added to the *Methods* section (page 12, line 564-574).

2. In the RBC labeling protocol, DiD dye is used at 50 mg/mL, and 3.2 μL is added to the RBC suspension. Is there any evidence of dye toxicity or impact on RBC viability/circulation time (e.g., comparing labeled vs. unlabeled RBC half-life in pilot studies)?

Reply: The DiD staining protocol used in our study was adapted and optimized based on previously published work from our group and others [1-2]. To assess the potential influence of DiD staining on RBCs, we further examined the stained cells under a commercial microscope. The majority of DiD-stained RBCs retained their characteristic biconcave disk morphology, indicating that the staining process does not substantially induce structural damage. A representative brightfield image of DiD-stained RBCs has been added as Supplementary Fig. 11a. It is also important to note that the DiD-stained RBCs constitute only a small fraction (~0.017%) of the total circulating RBC population, minimizing any potential systemic toxicity or measurable impact on overall blood health or circulation dynamics in the mouse model. The calculation of the ratio of stained RBCs to native RBCs has been added to the *Methods* section (page 11, line 525-528).

With respect to RBC viability/circulation time, previous studies report that the half-life of normal RBCs in mice is ~22.6 days [3]. Direct measurement of the *in vivo* half-life of DiD-labeled RBCs was not feasible in the present study due to the lack of an animal license for longitudinal experiments (required for mice with long-term cranial windows). As an indirect assessment, we quantified the number of detectable labeled RBCs over ~25-min observation window using our imaging system (Supplementary Fig. 11b). A simple linear regression fit revealed a slow decrease trend (clearance rate: -43.41 cells/s), suggesting no acute destabilization or rapid clearance of the labeled RBCs under the experimental conditions. Moreover, our functional imaging experiments were completed within a 4.5-min acquisition window, during which the number of DiD-stained RBCs can be considered stable.

The corresponding descriptions have been added to the *Methods* section (page 11, line 523-531).

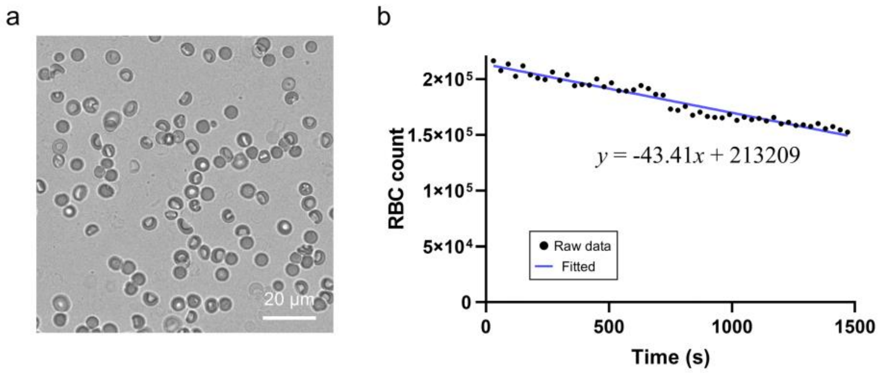

Supplementary Figure 11. Assessment of DiD-stained RBC integrity and stability.

References:

[1] Zhou, Quanyu, *et al.* "Cortex-wide transcranial localization microscopy with fluorescently labeled red blood cells." Nature Communications 15.1 (2024): 3526.

[2] Zhang, Zhenkun, *et al.* "Imaging volumetric dynamics at high speed in mouse and zebrafish brain with confocal light field microscopy." Nature biotechnology 39.1 (2021): 74-83.

[3] Dholakia, Urshulaa, *et al.* "Determination of RBC survival in C57BL/6 and C57BL/6-Tg (UBC–GFP) mice." Comparative medicine 65.3 (2015): 196-201.

3. The manuscript mentions that mice were euthanized with ketamine overdose post-imaging. Please confirm that the ketamine dose complies with the Swiss Federal Act on Animal Protection (e.g., dose per gram of body weight) and provide this detail for transparency.

Reply: We apologize for the confusion. The mice used in this study were euthanized under deep isoflurane anesthesia (5%) for 5 min followed by cervical dislocation. We confirmed this procedure complies with the Swiss Federal Act on Animal Protection. The corresponding description has been updated in the *Methods* section (page 11, line 553-554).

Typos and Language:

The manuscript is well-written, but a few minor corrections are suggested:

Page 2, Line 74: "depth-retrival uncertainty" should be "depth-retrieval uncertainty".

Reply: Thank you for pointing out the typo. It has been corrected to "depth-retrieval uncertainty" in the revised manuscript (page 2, line 76).

Page 5, Line 257: "This phenomenon has predominantly manifested" could be rephrased to "This phenomenon was predominantly observed".

Reply: The sentence has been revised as suggested (page 6, line 278).

Reviewer #2

In the manuscript the authors present a novel mesoscopic fluorescence imaging platform which is based on a double-helix phase mask finalized at performing high-speed volumetric imaging of cerebral and calvarial vasculature structures. Double-helix point spread function imaging has been already proposed in microscopy imaging, but here the authors very convincingly extend the technique into the mesoscopic area providing detailed guidance on techniques and strategies for concequent artifacts removal.

I think that the possibility to perform (as abundantly shown here) in-vivo depth-resolved large-area imaging at the capillary scale through the mouse intact skull is a game-changer of extremely great relevance and offers great potential for probing cerebrovascular alterations as here indicated. It could also find extended use in the cardiovascular field I would imagine.

The manuscript is extremely well written. A large amount of carefully given details is present, and it's very clear in its content, description, and purpose. I found both the exposition and the methodology very clear. Also the language is pretty fluent. The supplementary information is also extremely well done.

Data are very nicely collected and the images appear to be of great quality. I was greatly impressed by the amount of work the authors spent not only in the system characterization but also on how extensively they have tried to demonstrate its use in the biological imaging context.

Reply: We thank the Reviewer for the positive evaluation and constructive suggestions. Please find below our answers to the specific comments.

As a minor thing I would explain better the classical double helix point spread function strategy in the introductory section in order to better set a basis for what comes next in the paper. Probably not everybody if familiar with it.

Reply: We thank the Reviewer for the valuable suggestion. As suggested, we have added a brief description of the original application and basic performance of double-helix point spread function (DH-PSF) in super-resolution microscopy to the *Introduction* section (page 2, line 64-67).

Regarding the figures they are all excellent in both quality and content description.

In Figure 1, in panel "b" it is a little bit difficult to see clearly what is going on.

Reply: We apologize for the confusion. Fig. 1b compares DH-PSF intensity patterns at different axial positions before (top row) and after (bottom row) applying the optimization algorithm. To better highlight the improvement in the intensity of PSF after optimization, we intentionally used different intensity scale ranges for the two rows (0-0.3 before optimization, 0-1.0 after optimization). For clarity, we have revised the figure legend to explicitly state the distinct intensity scales used for the top and bottom rows of Fig. 1b (page 16, line 754-756).

Panel "h" indicates a later resolution of 8.76 microns. I would guess this is calculated on one fitted bead. It would be probably better to analyze on more RBCs?

Reply: In the original manuscript, the lateral resolution was calculated from a single bead. To provide a more representative assessment, we have randomly selected n = 15 RBCs across the field of view (FOV) for lateral resolution estimation. The analysis yields a lateral resolution of 8.61 ± 1.41 μm (mean ± SD), as shown in the updated Fig. 1h (page 16, line 761-762).

Reviewer #3

This manuscript presents a new approach to mesoscopic fluorescence imaging by introducing a high-efficiency double-helix point spread function (DH-PSF) scheme for real-time, depth-resolved microangiography in the intact mouse brain. The work addresses a long-standing challenge in neuro-imaging, namely achieving rapid, volumetric visualization of large-scale cerebrovascular networks without compromising spatial resolution, field-of-view, or functional sensitivity.

Conventional two-photon and localization-based optical methods offer either high spatial resolution or functional flow measurements, but not both simultaneously at mesoscale dimensions. Ultrasound and optoacoustic localization methods provide volumetric access but at the expense of optical resolution, complexity, or transcranial performance. Point spread function (PSF) engineering has emerged as a promising strategy to achieve depth-resolved optical imaging, where axial information is encoded into the PSF shape, however, particularly DH-PSF has previously not been successfully extended to mesoscopic imaging due to low transmission efficiency and severe field-dependent aberrations.

The authors overcome these limitations through a re-designed, 18-mm-aperture, DH phase mask optimised via iterative phase retrieval. Notably, it yields a 3.25-fold gain in photon efficiency. The authors then use a spatially adaptive calibration method to reduce depth-retrieval error by over 8x. When this scheme was combined with multifocal scanning, the system achieved real-time (3 Hz) volumetric imaging over a large 6.6×6.6×0.8 mm^3 field at ~9×9×4 μm^3 resolution. This enabled reliable discrimination between skull and cortical vessels and quantitative mapping of perfusion dynamics. In widefield mode, the platform further provides 3D flow velocity and direction measurements by tracking labeled RBCs.

The ability for both morphological and functional readouts makes the proposed method a powerful tool, which the authors applied to a few compelling use cases. The system was able to visualize depth-resolved perfusion patterns, reconstructed full cortical flow topology, and revealed marked structural and functional abnormalities in glioma vasculature, including increased density, tortuosity, and bidirectional flow.

Overall, this manuscript introduces a powerful and potentially widely applicable imaging tool which can provide a notable advance for high-throughput mesoscopic neurovascular imaging. As such the manuscript could be a good fit to Nature Communications, however, further clarification and additional validations, as outlined in more detail below, are suggested. Addressing these points would greatly enhance the clarity and impact of the work and bring it closer to the standards expected for a multidisciplinary journal.

Reply: We sincerely thank the Reviewer for the positive and constructive evaluation of our work. In the revised manuscript, we carefully addressed the concerns from both technical and biological aspects. Specifically, we have provided detailed descriptions of spatial resolution, phase mask design, spatially adaptive calibration, and multifocal scanning parameters; a systematic analysis of the impact of photon count and noise levels on depth estimation accuracy; a comprehensive evaluation of the influence of fluorescent emitter density and scattering properties on the fidelity of ADMM reconstruction, and statistical analyses for biological findings. These clarifications are supported by additional experiments, simulations, and analyses. Please find below our point-by-point responses to the comments.

Technical comments:

-) I suggest the authors include more systematic quantitative comparisons (resolution, accuracy, imaging depth, speeds, etc) against other state-of-the-art mesoscopic imaging methods, perhaps as a Supplementary Table?

Reply: We fully concur. Accordingly, we have summarized the imaging performance of our approach alongside other state-of-the-art mesoscopic imaging modalities (i.e., multi-photon microscopy, optical coherence tomography, optoacoustic microscopy) in the new Supplementary Table 1. We have referenced Supplementary Table 1 in the *Discussion* section to emphasize the unique advantages of our platform for multi-functional neuroimaging (page 7, line 322-324).

-) How sensitive is DH-PSF performance to deviations in phase mask height profile or surface roughness? Has this been studied by the authors? Elaborating on this could be useful for further studies and for the general uptake of the technique.

Reply: As suggested, we investigated the sensitivity of the double-helix point spread function (DH-PSF) to deviations in the phase mask height profile using numerical simulations. Random surface-height perturbations with root-mean-square (RMS) amplitudes of 10 nm, 20 nm, 30 nm, 40 nm, and 50 nm were introduced to emulate typical fabrication errors. By calculating the maximum intensity of the PSF at various defocus planes and comparing them with the ideal error-free case, we observed negligible performance degradation even at 50 nm height errors (RMSE = 3.87%), as shown in the new Supplementary Fig. 2. These results indicate that our designed DH-PSF exhibits robustness to phase mask manufacturing errors. We have added the corresponding descriptions in the *Results* section (page 3, line 135-137).

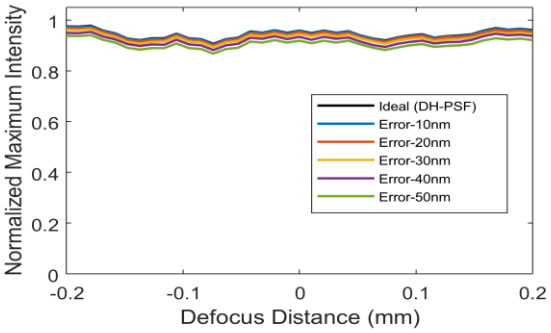

Supplementary Figure 2. Impact of phase mask manufacturing errors on the maximum intensity of DH-PSF across different depths.

-) Was photon efficiency measured across the entire FOV or only on-axis? How does transmission vary off-axis?

Reply: We thank the Reviewer for raising this point. Photon efficiency was measured across the entire field of view rather than only on-axis by performing a comprehensive, field-wide statistical analysis over the full 17 × 17 spot array (289 spots). To robustly capture the characteristic morphology of the DH-PSF, we adopted a global metric based on net integrated intensity for all spots, which provides a consistent and position-independent estimation of photon throughput.

As shown in Response Fig. 4a, the system without phase mask exhibits pronounced spot-to-spot intensity fluctuations, with notably reduced intensity in off-axis regions. In contrast, after introducing the DH phase mask, the spot intensity becomes lower but markedly more consistent across the field of view, as reflected by a reduction in the coefficient of variation from 23.05% to 18.35%.

To further quantify the relative photon transmission across the entire FOV, we computed the photon efficiency on a spot-by-spot basis as the ratio between the integrated intensities with and without the DH mask. The resulting spatial efficiency map is shown in Response Fig. 4b. While slightly higher efficiencies are observed in some peripheral regions, the overall efficiency distribution remains highly uniform across the FOV. The global analysis yields a mean photon efficiency of 0.544 with a CV of 6.90%, indicating minimal off-axis degradation and excellent spatial consistency.

The same photon efficiency calculation strategy was consistently applied in Fig. 1e and Fig. S3 (panel b). The corresponding descriptions have been added to the *Results* section (page 3, line 111-112).

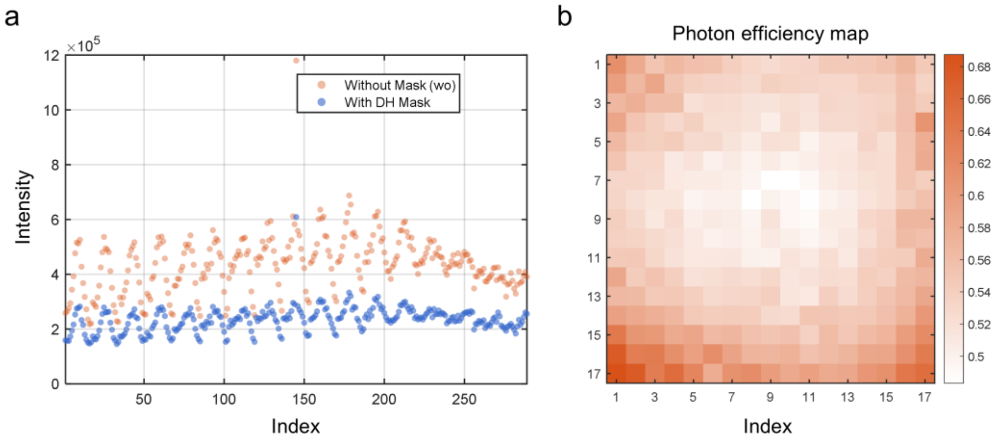

Response Figure 4. Quantitative evaluation of intensity and photon efficiency. a Spot-by-spot integrated intensity across the full 17 × 17 spot array with (blue) and without (orange) the DH phase mask. b Spatial map of photon efficiency, calculated as the ratio of integrated intensity with and without the DH phase mask.

-) How robust is depth estimation to noise and variations in photon counts (signal intensity)?

Reply: To systematically assess the impact of photon count (N) and noise level (read noise σ_{read} and background noise B_0) on depth estimation precision (σ_z), we conducted a three-phase Monte-Carlo simulation (Supplementary Note 1). We quantified σ_z across N ranging from 500 to 10000, σ_{read} ranging from 1 to 10 ADU, B_0 ranging from 10 to 500 ADU. The results (Supplementary Fig. 1) confirm the robustness of DH-PSF depth localization against varying noise characteristics: for photon count (N), precision significantly improved, dropping from an average σ_z of 9.19 μm (N =500) to 0.93 μm (N =10,000). Regarding camera noise, increasing σ_{read} from 1 ADU to 10 ADU (at fixed N =2000) resulted in a moderate increase in σ_z from 2.68 μm to 3.88 μm. Increasing B_0 across the full range (10-500 ADU) causes σ_z to degrade from 2.10 μm to 6.44 μm. Our results confirm that the DH-PSF method maintains high accuracy and scales its precision primarily according to theoretical limits, with background noise identified as the dominant practical limiting factor. The corresponding contents have been added to Supplementary Note 1, Supplementary Fig. 1, and the *Results* section (page 3, line 133-137).

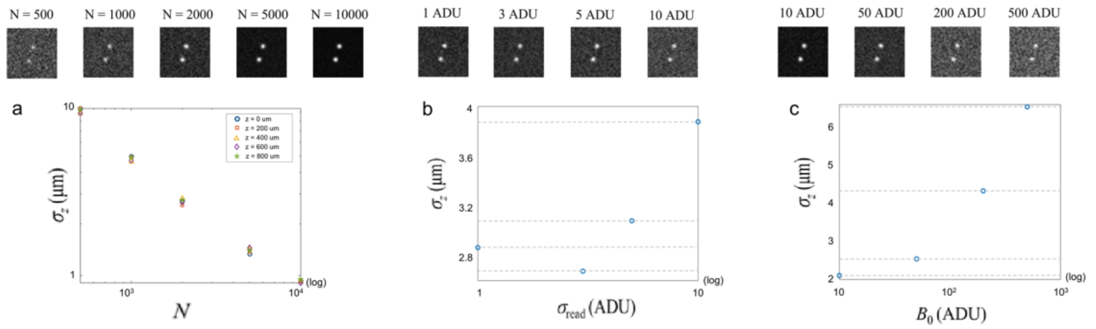

Supplementary Figure 1. Systematic analysis of factors influencing DH-PSF depth estimation precision. a-c Dependence of estimation precision (σ_z) on photon count (N), camera readout noise (σ_{read}), and background noise (B_0).

-) A quantitative characterization of skull-induced aberrations (e.g., wavefront error maps) would significantly strengthen the claim of robust transcranial performance.

Reply: As our system does not currently integrate a wavefront-sensing module, we are unable to directly measure skull-induced aberrations. Instead, we performed numerical simulations to evaluate their influence on DH-PSFs by introducing synthetic aberrations composed of selected Zernike modes [1-2]. The specific Zernike components and their relative weights (prior to final RMS scaling) are summarized in the table below (Response Table 1). Three aberration conditions were examined, with total wavefront error RMS values ranging from 2 radians (0.32 waves) to 8 radians (1.27 waves).

Calibration curves relating PSF orientation to depth were simulated using numerical convolution model under these three aberration conditions (Response Fig. 4). The orientation angles measured from the aberrated DH- PSF exhibited a minimal deviation from the ideal DH-PSF, yielding an $RMSE_\theta$ of 0.194°-0.619°. We further performed linear fitting on both the ideal and aberrated θ–z curves to estimate the aberration-induced focus shift. The resulting focus shift ranged from 1.10 to 3.44 μm.

Response Table 1: Zernike Mode Components for Aberration Simulation

Noll Index (j)	Zernike Term	Relative Amplitude (ΔA, λ)
2	Y-Tilt	0.50
3	X-Tilt	0.25
4	Defocus	0.10
5	Oblique Astigmatism	0.15
7	Y-Coma	0.10
8	X-Coma	0.20
9	Primary spherical	0.10
10-15	Higher-Order Aberrations	0.01 × randn(6, 1)

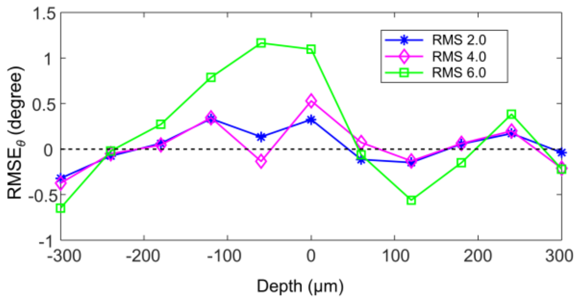

Response Fig. 4 DHPSF orientation angle error under different aberration severities.

References:

[1] Yonghyeon Jo et al., Through-skull brain imaging in vivo at visible wavelengths via dimensionality reduction adaptive-optical microscopy. *Sci. Adv*. 8, eabo4366(2022).

[2] Ji, N., Sato, T. R. & Betzig, E. Characterization and adaptive optical correction of aberrations during in vivo imaging in the mouse cortex. *Proc. Natl. Acad. Sci. U. S. A.* 109, 22–27 (2012).

-) Regarding the ADMM-based reconstructions and resolution claims: Under high emitter density, how often does the ADMM approach mislocalize overlapped PSFs? How does this perform in more scattering phantoms? Phantom experiment mimicking more realistic conditions of the mouse brain could help to establish realistic error rates.

Reply: We fully agree with the Reviewer that ADMM-based algorithms may underperform under conditions of high emitter density or strong scattering. To quantitatively assess these effects, we performed additional phantom experiments that mimic different emitter densities and scattering conditions. Specifically, fluorescent beads with a diameter of ~10 μm were immobilized in 0.65% agar to form a random 3D distribution of emitters, allowing PSF overlap to occur more generally, with emitters closely spaced both laterally and axially. The phantom was imaged using a widefield microscope equipped with DH-PSF sensing module. The same phantom was also imaged with the same widefield system using a high-NA objective (NA = 0.25) with z-scanning to render the 3D volume.

Different emitter densities were obtained by adjusting the ratio between fluorescent beads and agar, yielding final densities of 7.2×10^3/ml, 7.2×10^4/ml, and 3.6×10^5/ml (Supplementary Fig. 5). As particle density increased, more overlapping PSFs were observed. ADMM accurately recovered emitter locations at densities of 7.2×10^3/ml and 7.2×10^4/ml. At the highest density (3.6×10^5/ml), ADMM still reconstructed all bead positions; however, some incorrect localizations were observed (5/33 beads), especially in regions of PSF overlap. These errors are likely related to the difficulty in balancing the data-fidelity and TV regularization terms, causing the algorithm to overfit local intensity fluctuations or PSF overlaps.

To probe the impact of scattering, we modified the phantom by adding intralipid to the agar at final concentrations of 0, 0.6%, and 1.2% (Supplementary Fig. 6). In the absence of scattering (0%) or under low scattering (0.6%), ADMM reconstruction correctly recovered all bead localizations. Under increased scattering (1.2%), ADMM still detected all beads, although a small number of incorrect reconstructions were observed (2/9 beads). The reconstruction errors are expected to arise from two aspects: (1) the increasing scattering causes the practical PSF to deviate from the ideal PSF used for reconstruction, and (2) stronger scattering decreases the SNR of the acquired images.

Collectively, these results confirm the method's ability to resolve spatially and axially overlapping emitters over a practical range of emitter densities and scattering properties. The corresponding figures and descriptions have been added in Supplementary Fig. 5, Supplementary Fig. 6, and Supplementary Note 3.

-) How sensitive is axial velocity estimation to depth noise, especially given the ~4 μm axial uncertainty?

Reply: Given the 200Hz frame rate in our study, the axial localization uncertainty of ~4 μm corresponds to an axial velocity uncertainty of ~0.8 mm/s. Reported axial velocities in the murine brain range from 0 to 12 mm/s. For velocities on the higher end of this range, such uncertainty corresponds to ~6.7% relative deviation, while its impact becomes more pronounced for vessels exhibiting lower axial velocities. Consequently, improving axial velocity estimation will fundamentally require enhancing axial localization precision. These descriptions have been added to the *Results* section (page 5, line 212-217).

References:

[1] Tang, Jianbo, et al. "Improved color Doppler for cerebral blood flow axial velocity imaging." IEEE transactions on medical imaging 40.2 (2020): 758-764.

[2] Miller, D. R., et al. "Multi-modal microvascular cerebral blood flow velocity mapping with 14T single-vessel MRI and optical microscopy in the mouse brain." bioRxiv (2023): 2023-11.

Biological demonstrations and findings:
-) For the skull vs. brain vessel perfusion timing, is the observed separation consistent across all animals? Providing some statistics would strengthen the general findings, also for the other observations presented throughout Figs. 3-6.

Reply: We thank the Reviewer for the valuable suggestions regarding the statistical validation of our findings. Regarding the skull vs. brain vessel perfusion timing, the observed separation was consistent across all three mice imaged. To further validate this observation, we conducted a statistical analysis on n = 18 skull vessels and n = 18 brain vessels collected from three mice. The corresponding statistical analysis has been included in the revised Fig. 2j. A paired Student's t-test confirmed that brain vessels are perfused significantly earlier, exhibiting 4.22 ± 0.53 s lower time-to-peak (TTP) values.

As suggested, we have supplemented statistical analyses in Figs. 3-6. Specifically, we have added statistical analyses for both Vel_{2D} and Vel_{3D} across different vessel types (revised Fig. 3j). We also added a Supplementary Fig. 7 that provides expanded evidence by illustrating the differentiation of microvessels using axial flow dynamics and depth traces, showcasing multiple representative examples of varying vascular structures. And we have included the statistical results for vessel area fraction (VAF) and trajectory tortuosity (as shown in revised Fig. 5h and Supplementary Fig. 9).

-) Does the overall system performance differ significantly across animals? Has this been tested?

Reply: As suggested, we conducted a thorough evaluation of the system's performance, including lateral resolution and signal-to-noise ratio (SNR), across different mice and surgical preparations, and imaging systems. The lateral resolution was quantified by calculating the full-width-at-half-maximum (FWHM) of smallest resolvable vessels (n = 6 vessels). The SNR of DH-PSFs was calculated by averaging the SNR measured from n = 10 illumination spots (laser scanning system) and n = 10 stained RBCs (widefield system). The overall system performance was consistent, as summarized below.

Mouse strain	Number	Surgical procedure	Imaging system	Lateral resolution (μm)	SNR of DH-PSF
C57BL/6J (n = 6)	mouse 1	transcranial	Laser scanning	10.12 ± 0.35	2.02 ± 1.33
	mouse 2	transcranial	Laser scanning	9.86 ± 0.65	2.38 ± 1.24
	mouse 3	transcranial	Laser scanning	9.75 ± 0.26	1.81 ± 1.97
	mouse 4	cranial window	Widefield	8.98 ± 0.51	1.37 ± 1.03
	mouse 5	cranial window	Widefield	8.86 ± 0.46	1.24 ± 0.76
	mouse 6	cranial window	Widefield	9.05 ± 0.24	1.46 ± 0.83
Athymic Foxn1nu mice (n = 3)	mouse 7	skull thinning	Widefield	9.12 ± 0.27	1.77 ± 0.54
	mouse 8	skull thinning	Widefield	8.92 ± 0.34	1.89 ± 0.79
	mouse 9	skull thinning	Widefield	8.96 ± 0.44	1.82 ± 0.78

-) What is the minimal RBC labeling density required for stable flow reconstruction without excessive overlap?

Reply: In this study, ~2×10^6 DiD-labeled RBCs were injected into each mouse before imaging. Assuming a total blood volume of 1.5 mL for a 20 g mouse and an average RBC density of ~8.13×10^6 cells/µL [1-2], the labeled RBCs account for only ~0.017% of the total RBC population. This labeling density was empirically chosen to balance the temporal resolution required for vascular map reconstruction with the need to minimize PSF overlap.

Under these conditions, each frame contained ~100 detectable DiD-labeled RBCs within a 672×672 px sensor area, corresponding to an average inter-cell spacing of ~67 px (assuming approximately uniform spatial distribution). Given that each DH-PSF spans ~12 px (distance between the center of dual lobes), the probability of PSF overlap is low. To further mitigate potential artifacts from rare overlapping events, only DH-PSFs with high matching scores for interlobe distance, intensity symmetry, or angular-orientation correlation are retained for downstream reconstruction.

Reducing the labeling density would further decrease the probability of PSF overlap but would necessitate proportionally longer recordings to capture sufficient RBC trajectories and flow information for reconstructing a vascular map of comparable quality.

The corresponding descriptions have been updated in the Results section (page 4, line 185-189).

References:

[1] Leal, Joames KF, Merel JW Adjobo-Hermans, and Giel JCGM Bosman. "Red blood cell homeostasis: mechanisms and effects of microvesicle generation in health and disease." Frontiers in physiology 9 (2018): 703.

[2] Huisjes, Rick, et al. "Squeezing for life-properties of red blood cell deformability." Frontiers in physiology 9 (2018): 656.

Minor comments:

-) Could the authors provide more detail on computational requirements (processing time per volume) so one can assess the scalability for long recordings?

Reply: As suggested, we have added Supplementary Table 5, summarizing the processing time for each step of image reconstruction workflow in both the laser scanning and widefield microscope systems. We have added the corresponding descriptions in the *Methods* section (page 10, line 478-479).

Point-by-Point Response to the Reviewers' Comments

Reviewer #1

The authors have addressed most of my previous concerns through detailed explanations and substantial revisions to the manuscript. The additional data provided in the revised paper significantly strengthens the paper's conclusions.

However, a few minor points remain that require further clarification or technical refinement before the manuscript can be recommended for publication.

Data and Code Availability (Crucial for Reproducibility):

The manuscript relies heavily on custom algorithms (ADMM, K-means clustering) and a specialized phase plate design. To ensure the reproducibility of the findings and the utility of this platform for the broader imaging community, the authors should provide more than a "upon request" statement.

I suggest the authors deposit the core reconstruction scripts and a representative raw data sample (e.g., a short image sequence of the double-helix lobes) to a public repository like GitHub or Zenodo. At minimum, a detailed pseudo-code or a flowchart of the ADMM optimization process should be included in the Supplementary Information.

Reply: We thank the reviewer for this valuable suggestion regarding reproducibility and accessibility of the reconstruction workflow. In response, we have added a detailed pseudo-code for the double-helix image reconstruction pipeline in the Supplementary Information (Supplementary Algorithm 1). The ADMM implementation used in this study builds on the open-source DiffuserCam framework; we now cite the corresponding reference in the *Code availability* section (line 584, page 12) and include the GitHub repository link in the Reporting Summary. In addition, we have deposited representative raw data collected under both laser-scanning and wide-field illumination modes in Zenodo at: https://doi.org/10.5281/zenodo.18876905. The *Data Availability* section in the manuscript and Reporting Summary has been updated accordingly (line 579–581, page 12).

Reviewer #2

The authors replie to all the comments I had. I also found the replies to the other reviewers' comments very insightful.

Reply: We thank the Reviewer for the positive feedback on our revision and are sincerely grateful for the constructive suggestions, which have been very helpful in improving the quality of this work.

Reviewer #3

I have read the revised manuscript by the authors and found it much improved. My comments and questions were thoroughly answered and addressed. In particular, the more quantitative comparison as well as characterisation of the system performance in the presence of noise add valuable details. The additional experiments statistics for the animal experiments also strengthen the findings of the manuscript. I therefore recommend publication.

Reply: We thank the Reviewer for the positive feedback on our revision. We are grateful for the valuable suggestions in the last round, which have been very helpful in improving the quality of this work.
